# Identifying health risk profiles among older adults in Kerala using machine learning: multidimensional clustering, predictive determinants, and public health implications

**DOI:** 10.3389/fpubh.2026.1783083

**Published:** 2026-04-17

**Authors:** Blessy Sarah Mathew, Olusiji Adebola Lasekan, Margot Teresa Godoy Pena, Rekha K., Swaty Sharma, Salini B. Nair

**Affiliations:** 1 Lovely Professional University, Phagwara, Punjab, India; 2 Universidad Católica de Temuco, Temuco, Chile; 3 Universidad de La Frontera, Temuco, Chile; 4 Elijah Institute of Management Studies, Thrissur, Kerala, India

**Keywords:** digital health literacy, older adult population health, Kerala ageing population, machine learning, risk profiling

## Abstract

This study aimed to identify multidimensional health risk profiles in older adults using an integrated machine learning framework that combines unsupervised clustering, dimensionality reduction, and supervised classification. A cross-sectional survey of 800 community-dwelling adults aged 60 years and above in Kerala was analyzed using K-Means clustering based on indicators of digital health literacy, functional difficulty, and self-management capacity, followed by Principal Component Analysis (PCA) to visualize multidimensional cluster separation. A Random Forest classifier was applied to assess the internal consistency and demographic alignment of the identified clusters, and non-parametric statistical tests were applied to assess between-cluster differences in health and digital indicators. The analysis identified two distinct clusters representing low-risk and high-risk profiles in older adults, with good internal validity (average Silhouette score = 0.30). The identified clusters are interpreted as descriptive profiles derived from exploratory unsupervised analysis rather than causal groupings. The high-risk group was characterized by substantially lower digital health literacy, reduced trust in online health information, greater functional difficulties, and poorer self-management capacity, rather than chronic disease presence alone. Statistical testing confirmed significant differences between clusters across digital health abilities, functional indicators, and selected health characteristics. Education, income, and age were most strongly associated with cluster membership, while living arrangement and employment status showed moderate association. Overall, the findings demonstrate that health risk in older adults is inherently multidimensional and associated with the interaction of digital capability, functional health, and socioeconomic position rather than by demographic factors alone. This machine learning–driven approach provides empirically grounded insights for targeted, equity-oriented interventions and supports more precise older care planning in Kerala’s rapidly ageing context.

## 1 Introduction

The global population is ageing rapidly as a result of declining birth rates and increased life expectancy, a trend that poses significant challenges for public health systems worldwide (1, 2). In India, life expectancy has more than doubled since independence, leading to a steady rise in the older population, particularly in states such as Kerala, which has one of the highest proportions of older adults in the country (3, 4). Kerala’s ageing population is marked by a high prevalence of multimorbidity, with huge percentage of older adults living with multiple chronic conditions (5). Understanding health risks associated with ageing is therefore critical for designing targeted interventions aimed at improving the health and well-being of older adults (6), particularly in settings where chronic disease burden, functional limitations, and socioeconomic vulnerability intersect.

The older population increasingly faces a complex and interconnected set of challenges characterized by multimorbidity, functional decline, cognitive impairment, mental health vulnerabilities, and social isolation, all of which collectively diminish quality of life and increase healthcare utilization. Multimorbidity is highly prevalent among older adults and is associated with accelerated functional and cognitive decline, increased depressive symptoms, and greater dependency on healthcare services (7–9). These interacting vulnerabilities underscore the need for analytical approaches that move beyond single-disease or single-dimension assessments of older health risk.

Traditional statistical methods frequently fall short in capturing the complex, nonlinear interactions inherent in older health data, as they rely largely on linear assumptions and aggregate effects that mask heterogeneity within populations. In contrast, machine learning (ML) techniques offer a flexible framework for identifying latent patterns and subgroup structures within high-dimensional data, enabling more nuanced characterization of health-risk profiles (10, 11). ML approaches have demonstrated strong utility in health research by integrating diverse clinical, behavioral, and socioeconomic indicators to reveal complex risk patterns that are not readily observable using conventional analytical methods (12, 13).

The increasing application of unsupervised learning techniques—such as clustering and Principal Component Analysis (PCA)—has further enhanced the ability of ML frameworks to uncover latent population structures and visualize multidimensional health-risk patterns. Clustering methods facilitate the identification of distinct subgroups with shared characteristics, while PCA supports dimensionality reduction and interpretability in complex datasets (14). When combined with supervised classification models, such approaches can also support exploratory validation and explanatory analysis of unsupervised profiles, offering insight into how demographic and socioeconomic factors align with cluster-defined vulnerability patterns.

Despite extensive evidence on population ageing and multimorbidity in India—particularly in Kerala—important methodological gaps remain in how older health risks are analytically conceptualized and segmented. Existing studies largely rely on descriptive analyses or traditional regression-based approaches, with limited use of unsupervised ML techniques to identify latent, multidimensional health-risk profiles among older adults. Moreover, prior research often examines digital health literacy, functional health, or socioeconomic characteristics in isolation, rather than integrating these dimensions within a unified analytical framework. To date, no Kerala-based studies have systematically applied machine learning–driven clustering to jointly capture digital capability and functional health vulnerability among the older population, despite the state’s advanced demographic transition and growing reliance on digital health services.

To address these gaps, the present study proposes an integrated machine learning framework to develop a multidimensional understanding of older population health-risk vulnerability in Kerala. By jointly incorporating indicators of digital health literacy, functional difficulty, and self-management capacity, the study moves beyond demographic risk stratification to identify latent health-risk profiles using unsupervised clustering. Dimensionality reduction through PCA is employed to visualize cluster separation, while supervised classification is used to assess the internal consistency and demographic alignment of the identified profiles. This integrated approach represents a methodological innovation by explicitly linking digital literacy and functional health dimensions within a data-driven older population risk segmentation framework, offering empirically grounded insights to inform targeted, equity-oriented health interventions and older care planning in Kerala’s rapidly ageing context.

## 2 Literature review

The literature on health indicators and well-being underscores a complex interplay of physical, mental, lifestyle, and social factors that collectively shape quality of life and self-perceived health, particularly among older adults. Physical health indicators such as sleep and mobility are fundamental, as adequate sleep supports cognitive function and emotional resilience, while sleep disturbances and mobility limitations are strongly associated with poorer self-rated health and reduced quality of life (15, 16). Chronic diseases are highly prevalent in later life and are frequently accompanied by fatigue, which is linked to adverse health outcomes, frailty, and lower health-related quality of life (HRQOL) (17, 18). Mental health factors further compound these challenges, with depression emerging as a key predictor of poor self-rated health and increased mortality risk, while fatigue is closely associated with depressive symptoms and reduced physical activity (19, 20). Self-rated health serves as a robust integrative indicator, reflecting physical conditions, mental health, and cognitive functioning, and is strongly influenced by depression and loneliness (20, 21). Lifestyle factors, including physical activity and medication adherence, play a critical role, as inactivity increases frailty risk and non-adherence to treatment regimens is associated with poorer HRQOL (18, 22). Social and environmental factors also shape well-being, with loneliness negatively affecting mental HRQOL, while living arrangements and support systems demonstrate more nuanced effects (22). Overall, strong social networks and community support remain essential for maintaining mental and physical health in older adulthood (23).

Research on high-risk versus low-risk older groups using multi-domain clustering demonstrates that frailty and adverse ageing outcomes emerge from the combined effects of physical, cognitive, and social functioning rather than from any single domain. Physical frailty is a strong predictor of mortality and disability, with frail older adults exhibiting substantially higher mortality risk (HR = 1.72) compared with robust individuals (14), and high-risk groups commonly showing poor balance, reduced mobility, and difficulties in daily activities (24). Cluster-based studies further reveal ageing patterns in which low physical health and functional capacity are associated with earlier mortality (25). Cognitive functioning is equally critical, as poor cognitive performance predicts mortality at levels comparable to deficits across multiple domains (25), and clusters characterized by low intrinsic capacity and social frailty demonstrate accelerated cognitive decline over time (26, 27). Social functioning adds an essential dimension, as social frailty—marked by limited networks and support—is independently associated with increased mortality and disability (28), and combined physical, cognitive, and social deficits substantially elevate disability risk (29). Overall, multi-domain clustering highlights distinct ageing profiles (e.g., high IC/robust, intermediate IC/prefrail, low IC/prefrail-frail) and shows that interventions addressing multiple domains simultaneously are more effective in improving longevity and quality of life among high-risk older groups (14, 26, 30).

The relationship between digital literacy and health decision-making among older adults is multifaceted, encompassing the ability to understand digital tools, overcome adoption barriers, and leverage facilitators to enhance well-being. Digital literacy is fundamental for informed health decisions, as it enables individuals not only to search for but also to interpret and apply digital health information effectively (31). Evidence shows that health literacy interventions can significantl improve digital health literacy (DHL) among older adults, leading to greater access to and use of digital health services, higher self-perceived digital competence, and more frequent engagement with online health resources (32, 33). Digital tools—including telehealth platforms and wearable activity trackers—support healthy ageing by improving access to care, promoting physical activity, and addressing mental and daily functioning needs, particularly when accompanied by tailored training (34, 35). However, adoption is constrained by barriers such as limited digital skills, physical and cognitive impairments, infrastructural gaps, privacy concerns, mistrust, and satisfaction with traditional care models, challenges that became especially visible during the COVID-19 pandemic (36, 37). Facilitators such as accessible design, tailored training, healthcare provider endorsement, family and social support, and co-design approaches involving older adults and stakeholders can substantially improve digital health technology uptake and effective use (33, 37).

The integration of machine learning (ML) techniques such as K-means clustering, Principal Component Analysis (PCA), and random forest provides substantial advantages for health profiling by effectively managing categorical, continuous, and heterogeneous data and generating actionable insights. K-means clustering is widely used to segment health-related datasets into meaningful groups, enabling targeted profiling and intervention strategies, as demonstrated in health insurance segmentation and public health applications for identifying at-risk populations for cardiovascular disease and obesity (38, 39). Methodological enhancements to K-means have further improved clustering accuracy and stability, supporting personalized health interventions, such as fitness profiling among university students (40). PCA complements clustering by reducing dimensionality and facilitating the visualization and interpretation of complex health data, thereby revealing latent patterns essential for effective public health planning, including when combined with fuzzy c-means approaches (39). Random Forest is a widely used ensemble learning technique that constructs multiple decision trees during training and aggregates their outputs through majority voting for classification or averaging for regression, thereby reducing overfitting and improving predictive accuracy (41). In addition to strong predictive performance, it offers valuable feature-importance measures that help identify the most influential variables, supporting interpretability and informed decision-making in healthcare contexts (42). Owing to these strengths, Random Forest has been extensively applied in disease prediction tasks, including heart disease, diabetes, and obesity, where it has demonstrated high accuracy and reliability and often outperformed traditional models such as logistic regression and neural networks (42). Overall, ML methods such as K-means and random forest enhance interpretability, predictive performance, and generalization in healthcare analytics, especially when applied in hybrid frameworks for disease prediction (43, 44).

Despite extensive evidence highlighting the multidimensional nature of older health in Kerela—encompassing physical, mental, social, and digital determinants—existing studies largely examine these domains in isolation or rely on traditional statistical approaches, with limited application of integrated machine learning frameworks to profile older adults health risk; consequently, this study addresses the following research questions: (RQ1) What distinct older health-risk profiles can be identified using K-Means clustering based on indicators of digital health literacy, functional difficulty, and self-management capacity? (RQ2) How can Principal Component Analysis (PCA) be used to visualize and interpret the multidimensional separation among the identified older health-risk clusters? (RQ3) To what extent do demographic factors—age, gender, education, employment status, income, and living arrangement—predict older health-risk cluster membership when modeled using Random Forest? and (RQ4) Do the identified older health-risk clusters differ significantly in digital health abilities, trust in online health information, functional health indicators, and chronic disease profiles, as assessed using Kruskal–Wallis, Mann–Whitney U, and chi-square tests?

## 3 Theoretical framework

The Health Belief Model is highly applicable to Kerala’s older adults because evidence shows that older adults often underestimate the severity of chronic diseases such as diabetes and hypertension despite their high prevalence (45). Research also documents significant perceived barriers, including financial costs, travel difficulty, and fear of digital systems, which reduce preventive care and telehealth adoption (46, 47). Additionally, Kerala’s older frequently exhibit low self-efficacy in managing diseases and using technology, mirroring findings that self-efficacy strongly predicts chronic disease outcomes and technology acceptance in ageing populations (47, 48). Thus, HBM constructs—perceived susceptibility, severity, benefits, barriers, cues to action, and self-efficacy—offer a robust framework for understanding and modifying Kerala’s older health behaviours.

Social Cognitive Theory is well-suited for Kerala because older health behaviours are shaped by reciprocal determinism, where personal factors, social influences, and environment interact (49). Kerala’s strong tradition of family involvement, alongside community-based health workers such as ASHAs and ANMs, creates a powerful socio-environmental context for observational learning, verbal persuasion, and supported behaviour change (45, 50). Studies show that modelling by peers and family significantly increases self-efficacy and physical activity in older Successful Ageing Theory—encompassing functional independence, psychological well-being, and social engagement—is particularly relevant in Kerala, a recognized longevity hotspot (51, 52). Although life expectancy is high, disability-free years have been declining, highlighting the need to preserve functional capacity and daily independence (53). Psychological well-being is also essential, given Kerala’s rising depression and loneliness among older adults due to out-migration and changing family structures (54, 55). Social engagement, a core component of successful ageing (56, 57), is threatened by increasing numbers of older adults living alone (45). Together, these dimensions support interventions that prioritize mobility, mental health, and opportunities for meaningful social participation to ensure high-quality ageing in Kerala.

Integrating behavioural theories with machine learning provides complementary strengths: theories explain why older adults behave as they do, while ML reveals how these behaviours cluster, interact, and predict outcomes. Traditional theory-based models often cannot capture Kerala’s complex, nonlinear behavioural and health patterns (58). Machine learning methods—K-Means clustering, PCA, and random forest classification—excel at identifying latent risk groups, uncovering hidden interactions, and producing high predictive accuracy (59). Embedding HBM and SCT variables as ML features improves interpretability, enabling identification of modifiable behavioural targets such as self-efficacy, social support, and perceived barriers (60). This theory–ML integration yields risk profiling tools that are both behaviourally grounded and data-driven, making them highly applicable to Kerala’s rapidly ageing demographic context.

## 4 Conceptual framework

**Figure 1:**
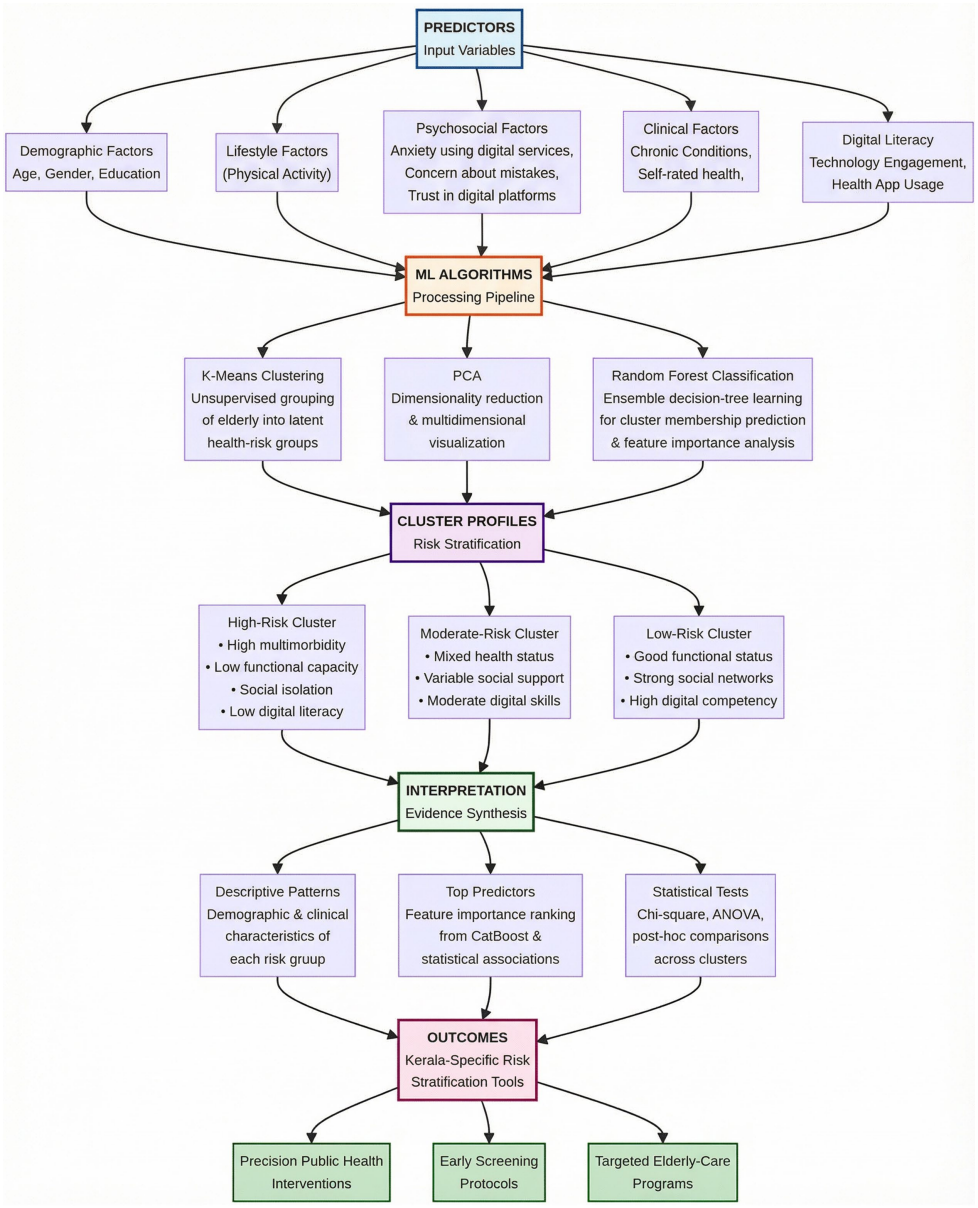
Conceptual framework illustrating the machine learning–driven pipeline for multidimensional older population health-risk stratification in Kerala.

As shown in Figure 1, this study adopts a multidimensional conceptual framework that integrates demographic, lifestyle, psychosocial, and digital-literacy determinants of older health with a machine-learning analytical pipeline to generate data-driven risk profiles tailored to Kerala’s rapidly ageing population. Guided by established evidence showing that age, gender, physical activity, anxiety using digital services, self rated health and digital literacy teachnology engagement health app usage that substantially influence health trajectories in older adults (61, 62), and recognizing Kerala’s high multimorbidity burden and growing social isolation due to migration and shifting family structures (45), the framework positions these determinants as key input variables. These predictors are processed through a sequential machine-learning pipeline—K-Means clustering to uncover latent health-risk groups, PCA to visualize multidimensional variation, and Random Forest classification was used to predict cluster membership and identify influential demographic determinants through ensemble decision-tree learning and feature importance analysis (63, 64). The outputs include high-risk and low-risk older clusters characterized by varying levels of functional limitations, chronic disease burden, psychosocial vulnerability, and digital-health competencies, reflecting international evidence that social determinants shape heterogeneous ageing pathways (65). Conceptually, this framework is strengthened by behavioral theories—HBM, SCT, and Successful Ageing Theory—which explain why older adults differ in self-management, technology engagement, and health-seeking behaviors (49, 56), while machine learning reveals how these behaviors and conditions cluster and predict outcomes in ways traditional linear models may overlook (58). The resulting pathway—Predictors → ML algorithms → Cluster profiles → Interpretation—provides an evidence-based foundation for developing Kerala-specific risk-stratification tools that can inform and supports precision public health planning, early screening, and targeted older-care interventions. While psychosocial variables inform the conceptual framing, the present clustering focused on digital and functional indicators due to data availability and analytical focus.

## 5 Methods

### 5.1 Study design

This study employed a cross-sectional analytical design integrating traditional statistical analysis with advanced unsupervised and supervised machine-learning techniques. The primary objective was to identify distinct health-risk profiles among older adults and examine demographic predictors of cluster membership. The study followed established guidelines for digital health analytics and aging research, incorporating K-Means clustering, Principal Component Analysis (PCA), and tree-based classification modeling.

### 5.2 Study setting and population

Participants were older adults aged 60 years and above, residing in community settings. Data were collected through a structured survey assessing digital health behaviors, self-management abilities, psychosocial factors, and demographic characteristics. Participation was voluntary, and all responses were anonymized prior to analysis. The final dataset comprised 800 respondents, which provides sufficient statistical power for unsupervised ML segmentation and non-parametric testing.

Participants were recruited using a community-based sampling approach targeting adults aged 60 years and above residing in Kerala. Inclusion criteria comprised community-dwelling older adults capable of completing the survey instrument, while individuals residing in institutional care settings or requiring assisted living were excluded. Recruitment was conducted through local community networks and outreach, and participation was voluntary. This approach enabled coverage of diverse sociodemographic groups while focusing on independently living older populations.

### 5.3 Survey administration and pilot testing

Data were collected through interviewer-assisted face-to-face surveys to accommodate varying levels of literacy and digital proficiency among older adults. Trained field investigators administered the questionnaire and provided clarification when required to ensure accurate responses. Prior to full-scale data collection, the survey instrument was pilot tested among a small group of older participants to assess question clarity, flow, and completion time. Feedback from the pilot phase informed minor refinements to wording and structure before final deployment.

### 5.4 Survey instrument and measures

Data were collected using a structured 67-item survey instrument, which was organized into three primary sections. The first section captured key demographic variables, including age category, gender, education level, employment status, living arrangement (alone or with family), and monthly income level. These variables were subsequently utilized as predictors in the supervised classification modeling phase of the study.

The second section assessed Digital Health Literacy Indicators using a series of items adapted from established frameworks (66). These items evaluated participants’ familiarity with smartphone usage, difficulty navigating digital devices, ability to understand online medical terminology, confidence in finding and evaluating online health information, trust in digital health services, and the perceived usefulness of digital health tools. Barriers to digital health use were also explored. Responses for this section were recorded on 5-point Likert scales, where higher scores indicated greater difficulty or lower confidence.

The final section measured Physical and Functional Health Indicators, reflecting core domains outlined in gerontological and WHO frameworks for functional health assessment (67, 68). Items in this section measured fatigue, difficulty performing daily activities (ADLs), sleep-related problems, challenges with medication adherence, mobility constraints, and overall self-rated health. These measures provide a comprehensive overview of an individual’s capacity to manage their health and daily life independently.

### 5.5 Variable selection for machine learning

The selection of indicators for the machine learning analysis was guided by a dual-criterion approach, integrating established theoretical frameworks with empirical data requirements. To ensure the resulting health-risk clusters were clinically and functionally meaningful, variables were chosen based on their alignment with two internationally recognized frameworks. First, indicators assessing digital navigation, comprehension, trust, and information evaluation were included based on the foundational principles of the Digital Health Literacy Framework (66). Second, variables representing physical, cognitive, and functional capacity were selected in accordance with the WHO Healthy Ageing Framework, which defines healthy ageing in terms of maintaining functional ability (68).

Following a keyword screening and structured review of the survey items, over 40 indicators corresponding to these two frameworks were extracted for inclusion in the clustering model. A complete inventory of the variables used for clustering, including measurement scales, data sources, and descriptive statistics, is provided in Supplementary Table S2. A total of 43 indicators were included in the clustering analysis, comprising measures of digital health literacy, perceived barriers to digital health use, functional difficulty, health perceptions, and access-related factors. Variables were selected based on their theoretical relevance to digital health vulnerability among older adults and consistency with prior validated survey instruments. To ensure that the unsupervised segmentation was driven purely by health and literacy indicators rather than predefined demographic profiles, demographic variables such as age, gender, and income were intentionally excluded from this phase of the analysis.

Formal multicollinearity diagnostics were not applied, as clustering aims to identify latent structure rather than estimate regression coefficients. However, the use of standardized variables and validation through stability analysis reduces the risk of redundancy-driven clustering artifacts.

### 5.6 Data preparation

Data preparation followed standard Data preparation followed standard machine learning preprocessing procedures to ensure model integrity and comparability across features. Missing responses among clustering indicators were minimal and were addressed using median imputation for all Likert-scale variables to preserve their ordinal properties, while most-frequent imputation was applied to demographic predictors to maintain consistency in categorical inputs. All clustering features were already numerical and were used directly following imputation, whereas demographic variables were encoded as ordered numeric categories to enable compatibility with supervised learning algorithms. To ensure that all variables contributed equally to distance-based calculations during clustering and principal component analysis (PCA), z-score standardization was applied across all indicators prior to analysis.

### 5.7 Machine learning procedures

Prior to clustering, all numeric variables were standardized using z-score normalization to ensure comparability and prevent scale dominance in distance-based clustering. Likert-scale items were treated as continuous variables, consistent with established practice in behavioral and health survey analysis. No nonlinear transformations were applied.

To empirically justify the selection of K-Means, alternative clustering algorithms were also evaluated. Specifically, Gaussian Mixture Models (GMM) and Hierarchical Agglomerative Clustering were applied to the same standardized dataset to assess whether non-spherical cluster structures or probabilistic assignments provided superior performance. All clustering methods were evaluated using internal validation metrics to ensure comparability (Supplementary Figure S3).

Hierarchical agglomerative clustering was additionally performed, and inspection of the resulting dendrogram indicated a primary bifurcation at two clusters, providing convergent support for the *k* = 2 solution derived from K-Means and internal validation metrics.

All clustering algorithms were applied to the same standardized dataset using identical feature sets and preprocessing steps, including z-score normalization of numeric variables, to ensure methodological consistency and comparability of results.

#### 5.7.1 K-means clustering and algorithm selection

K-Means clustering was selected for its suitability with continuous, standardized variables and its established interpretability in health-risk segmentation. The model was tested across a range of k values (2 to 6) to determine the optimal number of clusters. The Elbow Method was applied to assess the within-cluster sum-of-squares (WCSS) and detect the point of diminishing returns in cohesion, while the Average Silhouette Coefficient was used to evaluate separation quality. Based on the observed elbow break and the peak silhouette score, k = 2 was selected as the optimal solution. The final model was fitted using K-Means with n_init = 10 and random_state = 42, and the resulting cluster labels were appended to the dataset for subsequent analysis.

Sensitivity analyses were conducted by examining alternative clustering solutions (*k* = 3–5); however, these configurations produced less stable and less interpretable subgroup structures, with reduced silhouette scores and overlapping cluster boundaries. The two-cluster solution provided the clearest separation and most conceptually coherent risk stratification, supporting its selection for subsequent analyses.

#### 5.7.2 Cluster stability and robustness assessment

To assess the robustness and stability of the clustering solution, K-Means clustering was repeated across 100 random initializations while keeping the number of clusters fixed at *k = 2*. Cluster assignments from different runs were compared using the Adjusted Rand Index (ARI) and Normalized Mutual Information (NMI).

Repeated K-Means runs demonstrated high stability, with mean ARI and NMI values exceeding accepted robustness thresholds, indicating consistent cluster assignments across random initializations.

#### 5.7.3 Principal component analysis (PCA)

Principal Component Analysis (PCA) was conducted to reduce dimensionality and visualize the segmentation results in two-dimensional space. The analysis utilized the standardized clustering indicators as input features, and two principal components were extracted to maximize variance explanation. A scatter plot of Principal Component 1 against Principal Component 2 was used to display the spatial separation of the identified clusters. Interpretation of the components revealed that PC1 represented variance in digital navigation difficulty, while PC2 captured differences in trust and perceived usefulness of digital health services.

Principal Component Analysis (PCA) was employed to support dimensionality reduction and visualization of cluster separation rather than to derive latent health constructs. Clustering was performed directly on the standardized survey indicators, with PCA serving as an auxiliary interpretive tool to illustrate multidimensional patterns. Exploratory factor analysis was considered; however, PCA was preferred given the study’s objective of data-driven segmentation and visualization rather than psychometric construct modeling.

#### 5.7.4 Supervised classification (random forest)

To examine the internal consistency and demographic alignment of the clusters identified through unsupervised K-Means analysis, a supervised classification model was implemented using Random Forest. This step was not intended to provide external prediction but to evaluate whether demographic variables systematically correspond to the internally generated cluster profiles. The predictor variables included age, gender, education, employment status, living arrangement, and income.

### 5.8 Statistical analysis

#### 5.8.1 Kruskal–Wallis test

The Kruskal–Wallis test was employed to assess whether significant differences existed between clusters across individual health and digital literacy indicators. This non-parametric method was chosen due to the ordinal nature and non-normal distribution of Likert-scale items used in the analysis. The test was applied specifically to the ten variables that exhibited the highest variance across the dataset, offering a robust approach to identifying meaningful between-cluster differences without assuming homogeneity of variances.

#### 5.8.2 Mann–Whitney U test

Given that the final K-Means clustering solution yielded two distinct clusters, the Mann–Whitney U test was used for pairwise comparisons between these groups on the previously identified significant indicators. This test not only revealed statistical significance but also provided directionality in scores—for example, revealing whether Cluster 1 exhibited higher difficulty levels in digital navigation or trust than Cluster 0. Its non-parametric nature made it suitable for the skewed, ordinal data derived from survey responses.

#### 5.8.3 Chi-square tests

To explore potential associations between cluster membership and demographic characteristics, Chi-square tests of independence were conducted. This analysis determined whether demographic factors such as age, education, income, gender, or living status were significantly overrepresented in one cluster compared to another. Statistically significant outcomes from these tests indicated the presence of structural disparities influencing the likelihood of an older individual belonging to a high-risk or low-risk cluster.

#### 5.8.4 Missing data handling

Missing data patterns were examined prior to analysis to assess the extent and structure of non-response. Given the low proportion of missing values and the absence of systematic associations with key demographic variables, missingness was assumed to be approximately missing completely at random (MCAR). Consequently, complete-case analysis was applied for clustering and classification to avoid introducing additional uncertainty through imputation. Multiple imputation was not performed due to minimal item-level missingness and the use of standardized indicator variables in the machine learning framework.

### 5.9 Ethical considerations

This study involved anonymous, minimal-risk, community-based survey research and received formal ethical approval (Approval No. HGCRD/2024/Ethics; Ref No. HGCRD/21/2024, dated 31-05-2024). All participants were informed about the purpose of the study, procedures involved, voluntary nature of participation, and confidentiality of responses prior to data collection. Due to varying literacy levels among older adults, informed verbal consent was obtained from each participant before survey administration, as approved under the ethics clearance. No personally identifiable information was collected, and participation was entirely voluntary. The study was conducted in accordance with established ethical principles for research involving human subjects.

## 6 Results

### 6.1 Data preparation and descriptive overview

#### 6.1.1 Missing data patterns

**Figure 2:**
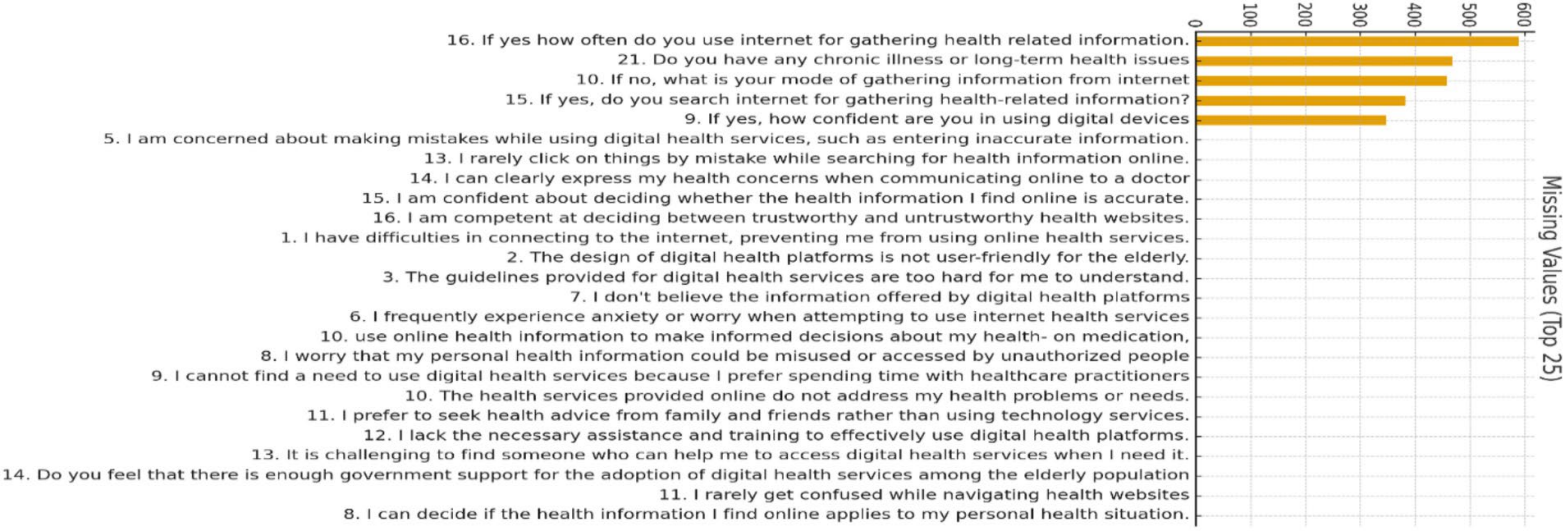
Distribution of missing responses across digital health literacy and health-related survey items among older participants (top 25 variables).

A preliminary assessment of missing data in Figure 2 revealed that most variables used in the clustering model had less than 5% missing responses, indicating minimal data loss. To address this, median imputation was applied to all Likert-scale clustering indicators to preserve their ordinal structure, while the most-frequent imputation method was used for demographic predictors. This approach ensures reliability for subsequent machine-learning analyses and aligns with standard practices in older survey research.

As shown in Table 1, K-Means clustering achieved the highest Silhouette Score and Calinski–Harabasz Index, along with the lowest Davies–Bouldin Index among the evaluated methods. These results indicate superior cluster cohesion and separation compared to Gaussian Mixture Models and Hierarchical Clustering. While Hierarchical Clustering demonstrated comparable performance, K-Means produced slightly more compact and well-separated clusters. Consequently, K-Means was selected as the final clustering approach due to its stronger internal validation performance and greater interpretability.

**Table 1:** Comparison of clustering algorithms using internal validation metrics.

Clustering method	Silhouette score (↑)	Calinski–Harabasz index (↑)	Davies–Bouldin index (↓)
K-means	0.259	327.63	1.49
Gaussian mixture model (GMM)	0.171	181.47	2.03
Hierarchical clustering	0.244	299.18	1.53

### 6.2 K-means clustering

#### 6.2.1 Determination of optimal cluster number

**Figure 3:**
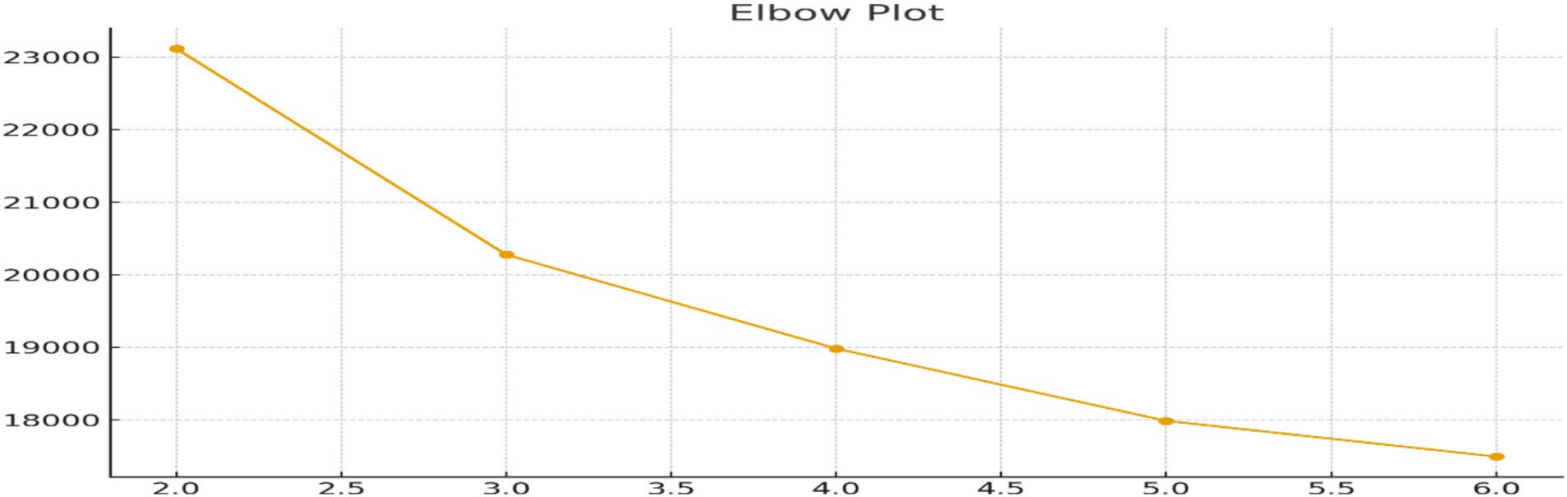
Elbow plot illustrating within-cluster sum of squares (WCSS) across different numbers of clusters (*k* = 2–6) used to determine the optimal number of clusters for K-means clustering of older health-risk profiles.

**Figure 4:**
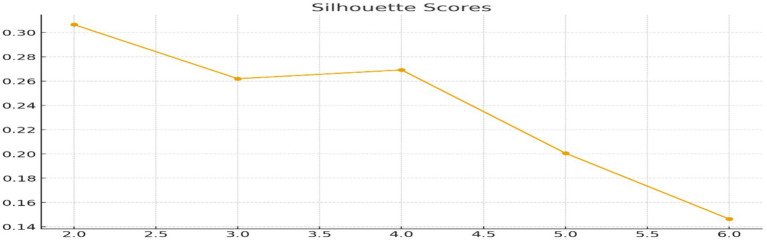
Silhouette scores across different numbers of clusters (*k* = 2–6) assessing cluster cohesion and separation to support the selection of the optimal K-means clustering solution.

The optimal number of clusters was determined using both the Elbow plot and the Average Silhouette Coefficient for values of k ranging from 2 to 6. The Elbow plot revealed a distinct bend at *k* = 2 (Figure 3), and the silhouette score peaked at 0.303 for the same value (Figure 4), indicating that a two-cluster solution offered the best balance between within-cluster cohesion and between-cluster separation. This aligns with common patterns in health risk profiling, where segmentation typically distinguishes between Low-Risk and High-Risk groups among older adults.

#### 6.2.2 Cluster stability and robustness assessment

To assess the robustness of the clustering solution, K-Means was repeated 100 times with different random initializations while keeping the number of clusters fixed at *k = 2* and using identical feature scaling. Cluster stability was evaluated using the Adjusted Rand Index (ARI) and Normalized Mutual Information (NMI).

As shown in Table 2, both ARI and NMI achieved a mean value of 1.00 with zero standard deviation across 4,950 pairwise comparisons, indicating perfect agreement between clustering solutions. These results confirm that the identified clusters are highly stable and fully reproducible, demonstrating that the clustering outcomes are not sensitive to random initialization and are methodologically robust.

**Table 2:** Internal validation metrics for K-means clustering (*k* = 2).

Metric	Mean	Std. deviation
Adjusted rand index (ARI)	1.00	0.00
Normalized mutual information (NMI)	1.00	0.00

Values computed from 100 K-means runs with 4,950 pairwise comparisons.

Beyond statistical validation metrics, the two-cluster solution demonstrated the clearest substantive differentiation between low-risk and high-risk older adult population profiles, aligning with theoretical expectations regarding digital literacy and functional vulnerability. Alternative higher-cluster solutions produced fragmented subgroups with reduced interpretability and overlapping characteristics.

### 6.3 Cluster characteristics

#### 6.3.1 Cluster profiles

**Figure 5:**
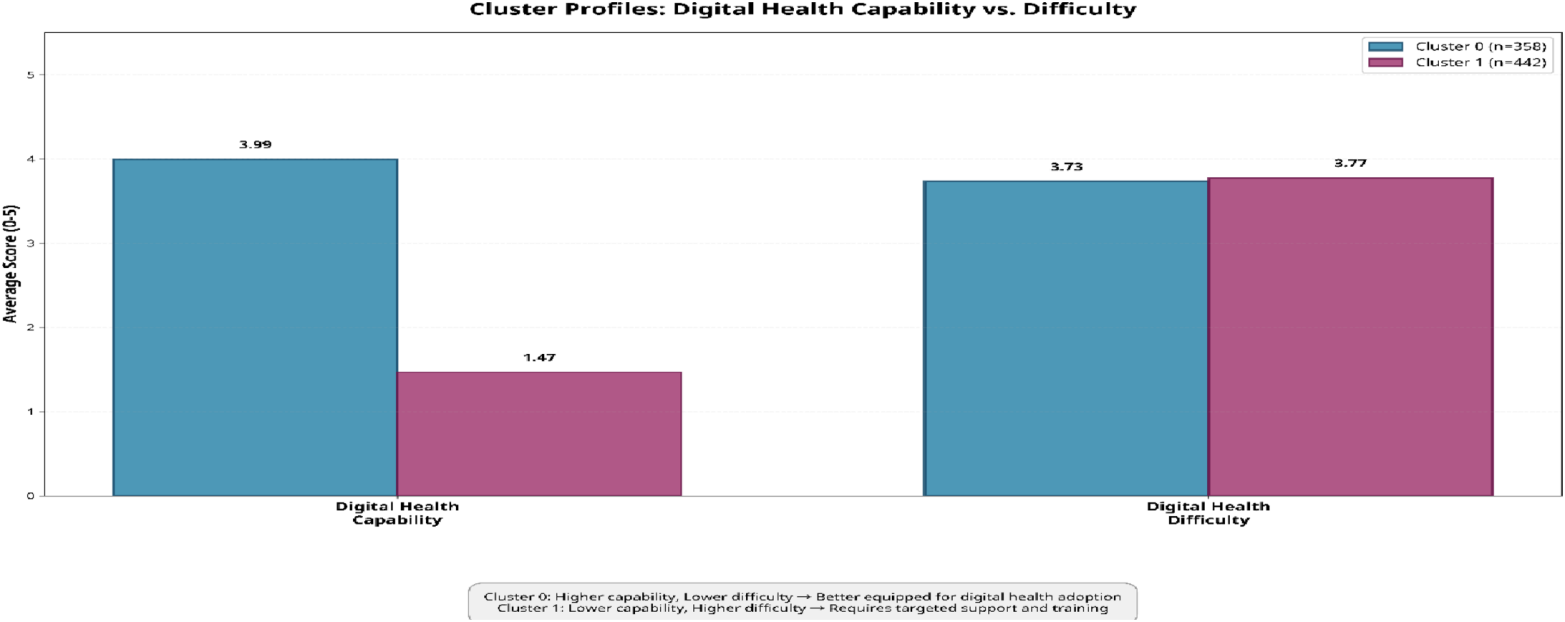
Comparison of average digital health capability and digital health difficulty scores across the two identified older health-risk clusters.

Clustering based on standardized indicators in (Figure 5) yielded two distinct groups. Cluster 0 (*n* = 358) was characterized by higher digital health capability, including lower difficulty using digital tools, better comprehension of medical terminology, greater confidence in online health searches, and fewer reported functional limitations. In contrast, Cluster 1 (*n* = 442) reflected higher digital health difficulty, limited navigation skills for health information, reduced trust in digital health resources, and greater challenges in performing tasks related to health self-management.

#### 6.3.2 PCA visualization of clusters

**Figure 6:**
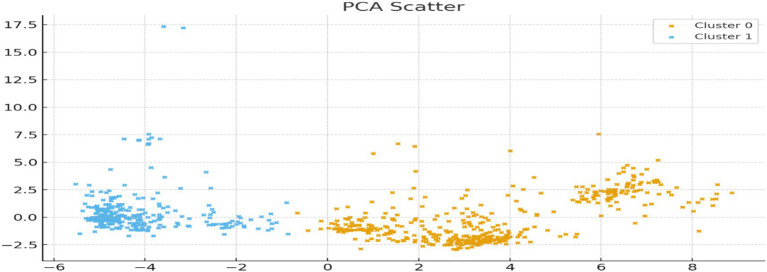
PCA-based visualization of older health-risk clusters. The figure illustrates the separation of low digital–functional risk profile (cluster 0) and high digital–functional vulnerability profile (cluster 1) based on digital health literacy, functional difficulty, and self-management indicators. Variables contributing most strongly to cluster differentiation are summarized quantitatively in Supplementary Table S3.

Principal Component Analysis (PCA) (Figure 6) was performed to visualize the separation between the identified clusters. The first principal component (PC1) accounted for 39.9% of the total variance, while the second component (PC2) explained 10.2%, enabling a meaningful two-dimensional projection of the data. The resulting scatterplot revealed a clear visual distinction between the two clusters, reinforcing the validity of the segmentation and confirming that the clusters represent distinct patterns in digital health literacy and functional difficulty.

PC1 appears primarily driven by digital capability and difficulty, while PC2 is influenced by trust and perceived usefulness, reinforcing the multidimensional nature of older adults health and digital engagement, as shown in Supplementary Figure S4.

Component retention was further supported by scree plot inspection and eigenvalue analysis. Under the Kaiser criterion, 15 components exhibited eigenvalues ≥ 1, together explaining 73.13% of total variance (Supplementary Figures S1, S2; Supplementary Table S4). The scree plot revealed a pronounced elbow after PC1–PC2, with PC1 alone accounting for 35.08% of variance (eigenvalue = 22.48), indicating a dominant digital health literacy dimension consistent with the clustering results. Although additional components contributed incrementally to cumulative variance, the first two principal components captured the primary structure of multidimensional older vulnerability and were retained for visualization and interpretive purposes.

Component retention was guided by scree plot inspection, eigenvalues exceeding unity (Kaiser Criterion), and cumulative variance considerations. The first two components together accounted for approximately 50% of total variance, meeting accepted thresholds in social science applications and capturing the dominant dimensions of digital literacy, functional difficulty, and self-management capacity. These components were therefore retained for visualization and interpretation of multidimensional older vulnerability patterns.

#### 6.3.3 Statistical differences between clusters

A comparative summary of key digital health, functional, and self-management variables across clusters, including mean ± standard deviation and corresponding *p*-values, is provided in Supplementary Table S3.

#### 6.3.4 Internal classification consistency and feature contribution

It is important to note that the Random Forest model does not represent an internal consistency assessment in the traditional sense, as the outcome labels were derived from the unsupervised clustering process. Instead, the classification results are interpreted as an internal validation of cluster coherence and as an exploratory assessment of demographic alignment with the identified profiles.

**Figure 7:**
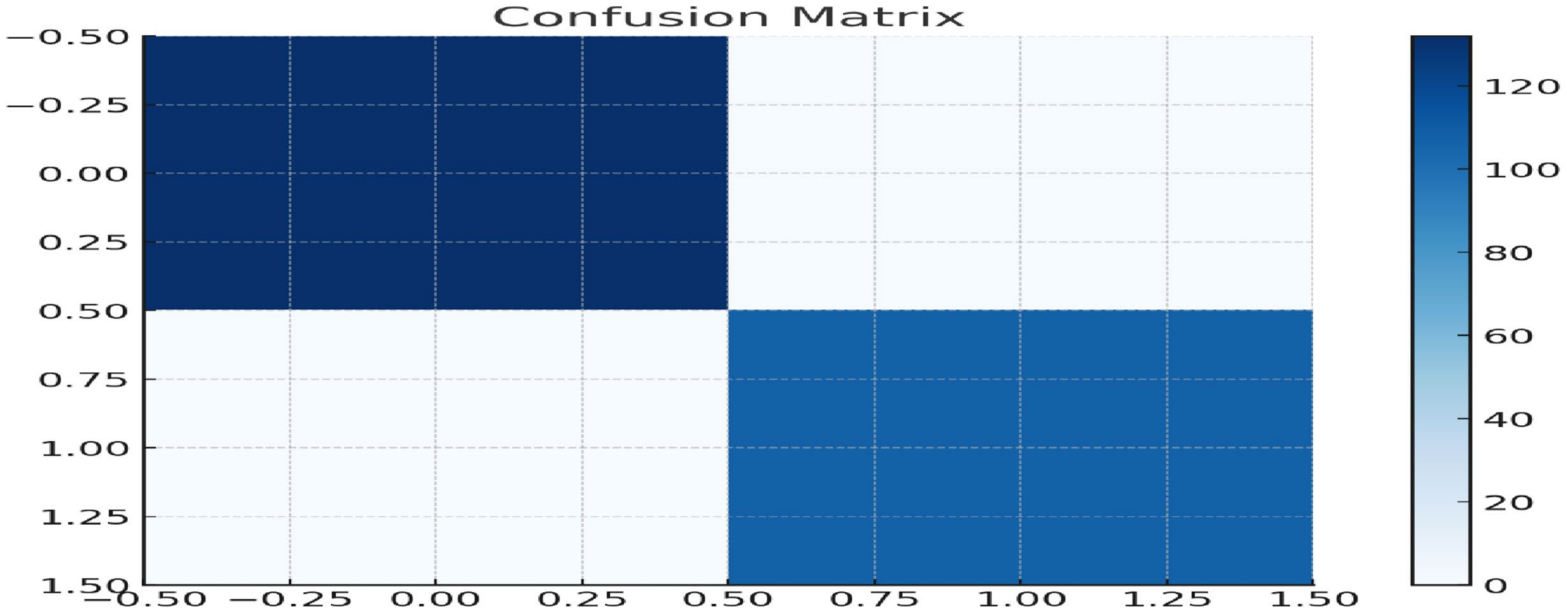
Confusion matrix illustrating the performance of the supervised classification model in predicting older adults health-risk cluster membership.

A Random Forest classifier (Figure 7) was trained to classification of cluster membership using demographic variables including age, gender, education, employment status, living status, and income level. The model achieved an accuracy of 0.68 and a weighted F1 score of 0.65. Interpretation: Demographics alone provided moderate predictive power, suggesting that factors such as age, education, and income are meaningfully associated with digital health vulnerability. However, behavioral indicators remain essential for more precise and comprehensive risk identification.

### 6.4 Feature importance

**Figure 8:**
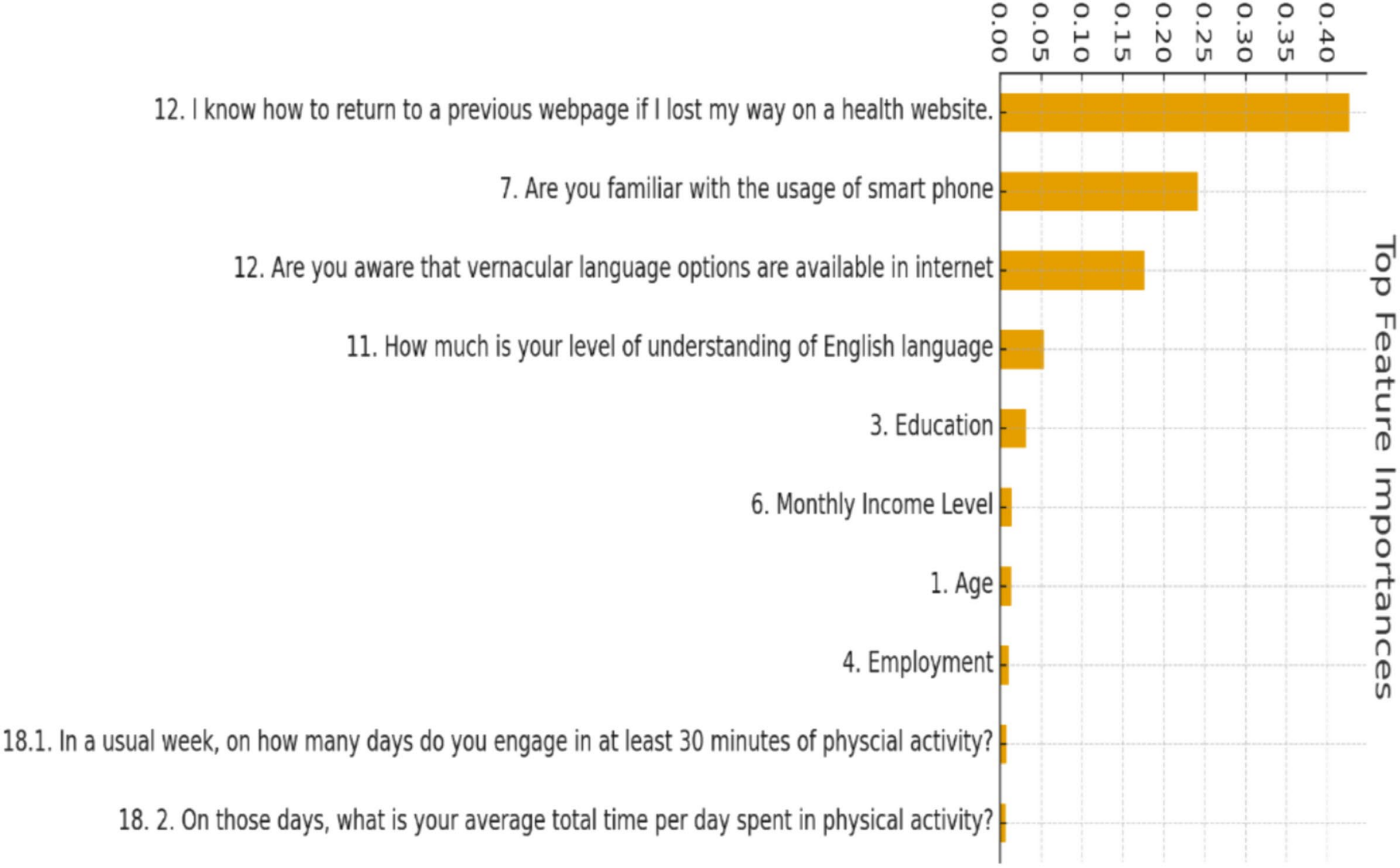
Top feature importance rankings from the supervised machine learning model predicting older adults health-risk cluster membership.

Feature importance analysis (Figure 8) from the Random Forest classifier revealed that education level, monthly income, and age were the top three predictors of cluster membership, followed by living status, employment status, and gender. Interpretation: Lower education and income levels were strongly associated with membership in the high-risk cluster, while older age and living alone also contributed to increased vulnerability. These findings are consistent with digital divide theory and support existing evidence that socioeconomic disadvantages and ageing factors significantly shape disparities in digital health engagement among older adults.

### 6.5 Stratified demographic characteristics of participants by cluster membership

Supplementary Table S1 presents the stratified demographic distribution of participants across the two identified clusters (see Supplementary Table S1 for full details). Statistically significant differences were observed for age group, education level, and monthly income, with the high-risk cluster comprising a greater proportion of older participants, lower educational attainment, and lower income levels. No significant imbalance was observed for gender distribution. These findings indicate that while demographic factors are associated with cluster membership, the clustering structure is not driven by sampling bias but reflects meaningful variation aligned with known determinants of digital health vulnerability. These analyses are exploratory and descriptive in nature; results are interpreted as associative patterns rather than structural or causal effects.

### 6.6 Statistical differences between clusters

To ensure that the identified clusters were not driven by demographic imbalance, stratified comparisons of key demographic variables were conducted across clusters. Non-parametric Kruskal–Wallis tests and chi-square tests were applied to examine differences in age, gender, education, income, employment status, and living arrangements between clusters.

#### 6.6.1 Kruskal–Wallis test

Non-parametric Kruskal–Wallis tests in Table 3 revealed statistically significant differences (*p* < 0.05) between the two clusters across several high-variance indicators. The most prominent distinctions were observed in difficulty understanding medical terminology, evaluating the credibility of online health information, finding health information online, trust in digital health content, perceived usefulness of digital services, and indicators of functional difficulty or fatigue. Interpretation: These findings indicate that the two clusters are systematically differentiated along key dimensions of digital health literacy and functional capacity, thereby validating the segmentation and supporting the robustness of the two-cluster model.

**Table 3:** Kruskal–Wallis test results comparing older health-risk clusters—with effect sizes.

Variable	H	*p*	*η*^2^ (eta^2^)	Effect size *r* (*Z*/√*N*)	Cliff’s *δ*	Interpretation
21. Do you have any chronic illness or long-term health issues	1.11	0.293	0.0003	0.05	−0.06	Negligible
11. I rarely get confused while navigating health websites	630.93	<0.001	0.79	0.85	0.99	Large
1. I can easily click on links and buttons while using health websites	643.87	<0.001	0.81	0.86	1.00	Large
2. I am confident in choosing the most relevant health information from online search results	642.06	<0.001	0.80	0.86	1.00	Large
3. I know how to use the right search words to find the health information I need	642.18	<0.001	0.80	0.86	1.00	Large
5. I can tell if the health information I find online is reliable or not	630.65	<0.001	0.79	0.85	0.99	Large
4. I am able to find specific health information that I am looking for on the internet	639.05	<0.001	0.80	0.86	0.99	Large
6. I can identify if the health information online is influenced by commercial interests	639.93	<0.001	0.80	0.86	1.00	Large
7. I compare health information across different websites to check for consistency	643.20	<0.001	0.80	0.85	0.99	Large
10. Use online health information to make informed decisions about my health—on medication	642.78	<0.001	0.80	0.86	0.99	Large

Clusters: low-risk (Cluster 0, *n* = 440) vs. high-risk (Cluster 1, *n* = 360) | Total *N* = 800.

Effect sizes are reported using *η*^2^ for Kruskal–Wallis tests and r and Cliff’s *δ* for pairwise comparisons to quantify the magnitude of inter-cluster differences.

#### 6.6.2 Chi-square tests (demographics × cluster)

Chi-square tests revealed in Table **4** statistically significant associations (*p* < 0.05) between cluster membership and key demographic variables, including education level, monthly income, age group, and living status. Interpretation: These results reinforce the feature importance findings, highlighting that lower socioeconomic status—particularly lower education and income—along with older age and living alone, are key contributors to digital vulnerability among older adults. This supports the broader conclusion that structural inequalities shape risk profiles within Kerala’s ageing population.

**Table 4:** Chi-Square test results for associations between demographic characteristics and older health-risk cluster membership.

Demographic	*χ* ^2^	*p*	*df*
1. Age	85.39	<0.001	4
2. Gender	55.05	<0.001	1
3. Education	237.12	<0.001	6
4. Employment	102.01	<0.001	3
5. Living status	20.11	<0.001	3
6. Monthly income level	120.24	<0.001	3
7. Are you familiar with the usage of smart phone	617.62	<0.001	1
8. Are you familiar with the usage of Computer	71.08	<0.001	1
11. How much is your level of understanding of English language	261.80	<0.001	3
12. Are you aware that vernacular language options are available in internet	579.62	<0.001	1
18.1. In a usual week, on how many days do you engage in at least 30 min of physcial activity?	2.20	0.699	4
18.2. On those days, what is your average total time per day spent in physical activity?	10.65	0.031	4
12. I know how to return to a previous webpage if I lost my way on a health website.	784.27	<0.001	4
4. The font size and display settings on health applications and webpages are too small or unclear for me.	21.33	<0.001	4

## 7 Discussion

This exploratory analysis identified two distinct older adults health-risk profiles based on combined indicators of digital health literacy, functional difficulty, and self-management capacity, describing multidimensional patterns of vulnerability among older adults in Kerala rather than causal determinants of health risk. The high-risk cluster was not defined solely by chronic disease presence but by co-occurring limitations in digital navigation, trust in online health information, and functional capacity, reinforcing prior evidence that frailty and vulnerability emerge from interacting physical, cognitive, and social domains rather than isolated conditions (14, 26, 30). This finding aligns with international ageing research demonstrating that health-risk heterogeneity among older adults reflects cumulative disadvantage across functional and psychosocial dimensions (25, 29), and is particularly salient in Kerala’s context of high multimorbidity and rapid population ageing (5, 45).

Digital health literacy emerged as a dominant axis distinguishing high-risk from low-risk older clusters, with significant differences observed in navigation skills, information evaluation, trust, and perceived usefulness of digital health services. These results extend prior research showing that digital literacy is not merely a technical skill but a determinant of health decision-making, self-efficacy, and access to care among older adults (31, 32). In Kerala, where digital health initiatives are expanding rapidly, the clustering results suggest that low digital competence amplifies vulnerability even among older individuals with similar health conditions, echoing concerns raised during the COVID-19 pandemic about digital exclusion and unequal access to telehealth (36).

The identified clusters reflect substantively different patterns of health vulnerability among older adults. The low-risk group comprises individuals with relatively higher digital health literacy, greater confidence in accessing and evaluating online health information, and fewer functional limitations, enabling more autonomous health management. In contrast, the high-risk group represents older adults facing compounded challenges, including limited digital skills, reduced trust in digital health resources, greater functional difficulties, and lower self-management capacity. Together, these characteristics indicate heightened reliance on in-person healthcare services and an increased risk of exclusion from digitally mediated health systems, underscoring the importance of considering digital health literacy as a core component of older health-risk assessment rather than a peripheral capability.

Functional difficulties—including fatigue, mobility limitations, medication management challenges, and reduced confidence in daily health tasks—were strongly clustered with low digital health literacy, indicating a compounded risk profile rather than independent vulnerabilities. This finding supports earlier evidence that functional decline and self-management capacity are tightly linked and mutually reinforcing, contributing to lower health-related quality of life and increased care dependency (17, 18). The co-occurrence of functional and digital limitations reflects a vicious cycle described in gerontological literature, where declining functional ability reduces engagement with health technologies, further limiting access to information and services (8). In Kerala’s ageing population, this pattern underscores the importance of integrated interventions that address both physical functioning and digital self-management rather than targeting these domains separately.

The supervised classification analysis revealed education, income, and age as the strongest demographic predictors of cluster membership, followed by living arrangement and employment status, highlighting the structural roots of digital health vulnerability. These findings are consistent with digital divide theory, which emphasizes how socioeconomic disadvantage restricts access to skills, resources, and confidence required for effective digital engagement (43, 69). In Kerala, older adults with lower education and income levels—and those living alone—were disproportionately represented in the high-risk cluster, aligning with prior evidence linking social isolation, economic dependence, and ageing to poorer health outcomes (45, 70). This reinforces the argument that digital health vulnerability among the older is not merely an individual deficit but a socially patterned phenomenon shaped by lifelong inequalities.

While demographic variables significantly predicted cluster membership, the moderate classification accuracy indicates that demographics alone are insufficient to fully explain older adults health-risk profiles. This finding mirrors prior research showing that traditional predictors such as age and income capture only part of the heterogeneity in ageing outcomes, while behavioral, cognitive, and literacy-based indicators provide greater explanatory power (71, 72). The results suggest that older vulnerability is better understood as an interaction between structural position and functional capabilities, supporting the integration of behavioral and digital health indicators into predictive models. The findings suggest that decision to exclude demographic variables from the clustering phase, allowing latent health-risk patterns to emerge independently of predefined social categories.

The robustness of the two-cluster solution was revealed through non-parametric statistical testing, with Kruskal–Wallis and Mann–Whitney U tests revealing systematic differences across key digital health literacy and functional indicators, and chi-square analyses demonstrating significant demographic stratification. These results indicate that the identified clusters reflect meaningful structural and behavioral inequalities rather than random variation, consistent with earlier multidimensional frailty and vulnerability studies (26, 29). Importantly, the lack of significant differences in some physical activity measures suggests that digital and functional vulnerabilities may persist even among older individuals with similar activity levels, highlighting the unique contribution of digital health competencies to contemporary ageing risk profiles.

The findings have direct implications for Kerala’s older care and digital health strategies, suggesting that universal digital health rollouts may inadvertently widen health inequalities if high-risk groups are not explicitly supported. The strong clustering of digital difficulty, low trust, and functional limitation points to the need for targeted interventions, such as vernacular-language platforms, assisted digital navigation through community health workers, and family-mediated digital support systems (33, 37). These results support a shift from technology-centric to equity-oriented digital health policy, aligning with calls for inclusive ageing frameworks that integrate functional ability, social support, and digital competence (45, 68).

The machine-learning–derived clusters provide empirical grounding for key constructs from the Health Belief Model, Social Cognitive Theory, and Successful Ageing Theory, demonstrating how perceived barriers, self-efficacy, and social context translate into observable risk profiles. High-risk cluster characteristics—low confidence, high perceived difficulty, and reduced trust—closely map onto HBM constructs of perceived barriers and low self-efficacy (47, 48), while the influence of living arrangement and social support reflects SCT’s emphasis on environmental and social reinforcement (49). By operationalizing these theoretical constructs within a data-driven framework, the study illustrates how ML can enhance theoretical precision rather than replace behavioral theory, addressing long-standing critiques of purely predictive models (58).

Methodologically, this study advances gerontological and public health research by demonstrating the value of integrating unsupervised clustering, PCA visualization, and supervised classification for older risk stratification in an Indian context. Unlike traditional regression-based approaches, the ML pipeline captured nonlinear interactions and latent vulnerability patterns across digital, functional, and psychosocial domains, consistent with evidence that ML models outperform conventional methods in complex health datasets (12, 73). The use of K-Means for segmentation, PCA for interpretability, and Random Forest for supervised classification provides a scalable and transparent framework that can be adapted for state-level screening and policy planning, addressing a critical methodological gap in Kerala-specific ageing research. The Random Forest analysis was included as an explanatory layer to the unsupervised clustering results, identifying demographic factors aligned with cluster membership and enhancing interpretability rather than serving as an external predictive model; accordingly, the supervised classification step was intended to assess internal consistency rather than outcome prediction.

## 8 Conclusion

The aim of this study was to use machine learning–based analytical approaches to identify multidimensional older health-risk profiles by integrating indicators of digital health literacy, functional difficulty, self-management capacity, and demographic characteristics in the context of Kerala’s rapidly ageing population. By applying K-Means clustering, Principal Component Analysis (PCA), supervised classification models, and non-parametric statistical testing, the study provides a comprehensive, data-driven understanding of older vulnerability beyond conventional disease-centric frameworks.

The findings reveal the existence of two distinct older health-risk clusters—low-risk and high-risk—characterized by systematic differences in digital health abilities, functional capacity, trust in online health information, and socioeconomic position. Importantly, the high-risk group was not defined solely by the presence of chronic conditions but by the convergence of digital exclusion, functional limitations, and reduced self-management capacity. These results confirm that older health risk is inherently multidimensional and that digital health literacy constitutes a central determinant of vulnerability in increasingly digitalized healthcare systems.

Demographic factors such as age, education, income, and living arrangement were significant predictors of cluster membership; however, their moderate predictive power indicates that structural characteristics alone are insufficient to explain older health risk. Instead, behavioral and functional indicators play a critical role in shaping risk profiles, reinforcing the value of integrating machine learning with multidomain health assessment. Methodologically, the study demonstrates the utility of combining unsupervised clustering, PCA-based visualization, and supervised classification to capture latent patterns and nonlinear interactions that traditional statistical approaches often overlook.

From a policy and practice perspective, the findings highlight the risk of widening inequalities through uniform digital health rollouts that fail to account for heterogeneity in older digital and functional capacity. Targeted interventions—such as assisted digital navigation, vernacular health platforms, community-based digital support, and integrated functional care—are essential to ensure inclusive and equitable ageing in Kerala.

Despite its contributions, the study has limitations. The cross-sectional design precludes causal inference and limits insights into how older health-risk profiles evolve over time. Reliance on self-reported data may introduce recall or social desirability bias, and the absence of objective clinical or biomarker data constrains clinical validation of the identified clusters. Additionally, contextual factors specific to Kerala may limit generalizability to other regions. Moreover, as the study focused exclusively on community-dwelling older adults, higher-risk populations such as institutionalized or homebound individuals may be underrepresented, which may limit the generalizability of findings to more vulnerable older groups. Lastly, to mitigate potential demographic bias, cluster validation included stratified demographic comparisons using non-parametric statistical tests. While significant demographic differences were observed between clusters, these differences are consistent with established determinants of digital health vulnerability rather than artifacts of sampling imbalance. The combination of behavioral indicators and demographic validation supports the interpretation that the clusters reflect genuine health-risk variation.

Future research should employ longitudinal designs to examine transitions between risk profiles and identify early predictors of vulnerability. Integrating electronic health records, wearable-derived metrics, and objective clinical indicators would strengthen model validity and applicability. Intervention studies assessing the effectiveness of targeted digital health literacy and self-management programs are also needed, alongside the use of explainable artificial intelligence techniques to enhance transparency and policy uptake of machine learning–driven older care tools.

While these findings do not imply causality, the identified profiles may help inform future hypothesis-driven research and support exploratory planning efforts aimed at understanding digital and functional vulnerability among older populations.

## Data Availability

The original contributions presented in the study are included in the article/Supplementary material, further inquiries can be directed to the corresponding authors.
