# Supplementary material for: Identifying health risk profiles among older adults in Kerala using machine learning: multidimensional clustering, predictive determinants, and public health implications
Source: Front Public Health. 2026 Apr 17;14:1783083. doi: 10.3389/fpubh.2026.1783083 (PMC13133658; doi:10.3389/fpubh.2026.1783083)
Supplement: Supplementary file 1 [file Table_1.DOCX]

**Supplementary material**

# Stratified Demographic Table

## Interpretation

Table S1 below presents demographic stratification of participants across simulated clusters. Cluster-wise breakdowns show differential distributions in age, gender, education, income, and employment. These disparities highlight how socio-demographic variables intersect with digital health vulnerability, providing valuable insight for targeted interventions.

## Table S1: Demographic Stratification by Cluster

| Cluster | Age | Gender | Education | Income | 1 | 2 | 3 | 4 |
| --- | --- | --- | --- | --- | --- | --- | --- | --- |
| 0 | 1 | 1 | 1 | 1 | 0 | 0 | 0 | 3 |
| 0 | 1 | 1 | 2 | 1 | 3 | 0 | 0 | 2 |
| 0 | 1 | 1 | 3 | 1 | 5 | 1 | 0 | 5 |
| 0 | 1 | 1 | 3 | 2 | 3 | 0 | 0 | 1 |
| 0 | 1 | 1 | 3 | 3 | 3 | 0 | 0 | 0 |
| 0 | 1 | 1 | 4 | 1 | 10 | 3 | 0 | 3 |
| 0 | 1 | 1 | 4 | 2 | 3 | 0 | 0 | 1 |
| 0 | 1 | 1 | 4 | 3 | 0 | 1 | 0 | 0 |
| 0 | 1 | 1 | 5 | 1 | 5 | 0 | 0 | 2 |
| 0 | 1 | 1 | 5 | 2 | 4 | 0 | 0 | 1 |
| 0 | 1 | 1 | 5 | 3 | 3 | 0 | 0 | 0 |
| 0 | 1 | 1 | 5 | 4 | 1 | 0 | 0 | 0 |
| 0 | 1 | 1 | 6 | 2 | 1 | 2 | 0 | 0 |
| 0 | 1 | 1 | 6 | 3 | 1 | 0 | 0 | 0 |
| 0 | 1 | 1 | 6 | 4 | 0 | 1 | 0 | 0 |
| 0 | 1 | 1 | 7 | 4 | 0 | 1 | 0 | 0 |
| 0 | 1 | 2 | 1 | 1 | 0 | 0 | 8 | 0 |
| 0 | 1 | 2 | 2 | 1 | 0 | 1 | 6 | 2 |
| 0 | 1 | 2 | 3 | 1 | 2 | 2 | 12 | 1 |
| 0 | 1 | 2 | 3 | 2 | 0 | 1 | 2 | 0 |
| 0 | 1 | 2 | 4 | 1 | 0 | 0 | 16 | 1 |
| 0 | 1 | 2 | 4 | 3 | 0 | 1 | 0 | 0 |
| 0 | 1 | 2 | 5 | 1 | 0 | 1 | 8 | 2 |
| 0 | 1 | 2 | 5 | 2 | 0 | 0 | 1 | 0 |
| 0 | 1 | 2 | 6 | 1 | 1 | 0 | 2 | 0 |
| 0 | 1 | 2 | 6 | 2 | 0 | 1 | 0 | 0 |
| 0 | 1 | 2 | 6 | 3 | 0 | 1 | 0 | 0 |
| 0 | 1 | 2 | 6 | 4 | 1 | 0 | 0 | 0 |
| 0 | 1 | 2 | 7 | 3 | 0 | 1 | 0 | 0 |
| 0 | 2 | 1 | 1 | 1 | 0 | 1 | 0 | 5 |
| 0 | 2 | 1 | 2 | 1 | 2 | 0 | 0 | 5 |
| 0 | 2 | 1 | 2 | 2 | 1 | 0 | 0 | 0 |
| 0 | 2 | 1 | 3 | 1 | 4 | 1 | 0 | 3 |
| 0 | 2 | 1 | 3 | 2 | 1 | 0 | 0 | 0 |
| 0 | 2 | 1 | 3 | 3 | 1 | 0 | 0 | 0 |
| 0 | 2 | 1 | 4 | 1 | 5 | 1 | 0 | 5 |
| 0 | 2 | 1 | 4 | 2 | 0 | 0 | 0 | 1 |
| 0 | 2 | 1 | 4 | 3 | 0 | 2 | 0 | 0 |
| 0 | 2 | 1 | 4 | 4 | 0 | 0 | 0 | 1 |
| 0 | 2 | 1 | 5 | 1 | 0 | 0 | 0 | 2 |
| 0 | 2 | 1 | 5 | 2 | 2 | 1 | 0 | 0 |
| 0 | 2 | 1 | 5 | 3 | 0 | 1 | 0 | 0 |
| 0 | 2 | 1 | 6 | 1 | 0 | 1 | 0 | 0 |
| 0 | 2 | 1 | 6 | 3 | 0 | 2 | 0 | 0 |
| 0 | 2 | 1 | 6 | 4 | 0 | 2 | 0 | 0 |
| 0 | 2 | 2 | 1 | 1 | 0 | 0 | 6 | 3 |
| 0 | 2 | 2 | 2 | 1 | 0 | 0 | 9 | 1 |
| 0 | 2 | 2 | 3 | 1 | 0 | 0 | 14 | 1 |
| 0 | 2 | 2 | 3 | 2 | 0 | 0 | 1 | 0 |
| 0 | 2 | 2 | 4 | 1 | 0 | 2 | 8 | 2 |
| 0 | 2 | 2 | 4 | 4 | 0 | 1 | 0 | 0 |
| 0 | 2 | 2 | 6 | 1 | 0 | 1 | 0 | 0 |
| 0 | 2 | 2 | 6 | 2 | 0 | 1 | 0 | 0 |
| 0 | 2 | 2 | 6 | 3 | 0 | 2 | 0 | 1 |
| 0 | 2 | 2 | 6 | 4 | 0 | 1 | 0 | 0 |
| 0 | 2 | 2 | 7 | 2 | 0 | 1 | 0 | 0 |
| 0 | 3 | 1 | 1 | 1 | 1 | 0 | 0 | 4 |
| 0 | 3 | 1 | 2 | 1 | 1 | 0 | 0 | 1 |
| 0 | 3 | 1 | 2 | 2 | 1 | 0 | 0 | 0 |
| 0 | 3 | 1 | 3 | 1 | 1 | 1 | 0 | 2 |
| 0 | 3 | 1 | 3 | 2 | 0 | 0 | 0 | 1 |
| 0 | 3 | 1 | 4 | 1 | 3 | 1 | 0 | 1 |
| 0 | 3 | 1 | 4 | 2 | 1 | 1 | 0 | 0 |
| 0 | 3 | 1 | 4 | 4 | 0 | 1 | 0 | 0 |
| 0 | 3 | 1 | 5 | 1 | 0 | 0 | 0 | 2 |
| 0 | 3 | 1 | 6 | 2 | 0 | 1 | 0 | 0 |
| 0 | 3 | 1 | 6 | 3 | 0 | 3 | 0 | 0 |
| 0 | 3 | 1 | 6 | 4 | 0 | 1 | 0 | 0 |
| 0 | 3 | 1 | 7 | 1 | 0 | 0 | 0 | 1 |
| 0 | 3 | 2 | 1 | 1 | 1 | 0 | 6 | 5 |
| 0 | 3 | 2 | 1 | 2 | 1 | 0 | 0 | 0 |
| 0 | 3 | 2 | 2 | 1 | 0 | 0 | 8 | 2 |
| 0 | 3 | 2 | 3 | 1 | 0 | 1 | 7 | 1 |
| 0 | 3 | 2 | 4 | 1 | 0 | 1 | 1 | 0 |
| 0 | 3 | 2 | 6 | 4 | 0 | 2 | 0 | 0 |
| 0 | 4 | 1 | 1 | 1 | 1 | 0 | 0 | 1 |
| 0 | 4 | 1 | 1 | 2 | 1 | 0 | 0 | 0 |
| 0 | 4 | 1 | 2 | 1 | 2 | 0 | 0 | 1 |
| 0 | 4 | 1 | 3 | 1 | 0 | 0 | 0 | 1 |
| 0 | 4 | 1 | 3 | 3 | 1 | 0 | 0 | 0 |
| 0 | 4 | 1 | 4 | 1 | 0 | 1 | 0 | 1 |
| 0 | 4 | 1 | 4 | 3 | 0 | 1 | 0 | 0 |
| 0 | 4 | 1 | 5 | 1 | 0 | 0 | 0 | 1 |
| 0 | 4 | 1 | 5 | 3 | 1 | 0 | 0 | 0 |
| 0 | 4 | 1 | 6 | 3 | 0 | 1 | 0 | 0 |
| 0 | 4 | 1 | 6 | 4 | 0 | 1 | 0 | 0 |
| 0 | 4 | 1 | 7 | 4 | 0 | 1 | 0 | 0 |
| 0 | 4 | 2 | 1 | 1 | 0 | 0 | 4 | 4 |
| 0 | 4 | 2 | 2 | 1 | 1 | 0 | 1 | 1 |
| 0 | 4 | 2 | 3 | 1 | 0 | 0 | 0 | 1 |
| 0 | 4 | 2 | 4 | 1 | 0 | 0 | 0 | 1 |
| 0 | 4 | 2 | 5 | 1 | 0 | 0 | 0 | 1 |
| 0 | 4 | 2 | 6 | 2 | 0 | 1 | 1 | 0 |
| 0 | 4 | 2 | 6 | 4 | 0 | 1 | 0 | 0 |
| 0 | 5 | 1 | 1 | 1 | 1 | 0 | 0 | 1 |
| 0 | 5 | 1 | 2 | 1 | 0 | 0 | 0 | 2 |
| 0 | 5 | 1 | 2 | 2 | 0 | 1 | 0 | 0 |
| 0 | 5 | 1 | 2 | 3 | 0 | 0 | 0 | 1 |
| 0 | 5 | 1 | 3 | 1 | 0 | 1 | 0 | 2 |
| 0 | 5 | 1 | 4 | 1 | 0 | 0 | 1 | 0 |
| 0 | 5 | 1 | 4 | 2 | 0 | 1 | 0 | 0 |
| 0 | 5 | 1 | 5 | 1 | 0 | 0 | 1 | 1 |
| 0 | 5 | 1 | 6 | 3 | 0 | 1 | 0 | 0 |
| 0 | 5 | 2 | 1 | 1 | 0 | 1 | 6 | 5 |
| 0 | 5 | 2 | 1 | 2 | 0 | 0 | 0 | 1 |
| 0 | 5 | 2 | 2 | 1 | 0 | 1 | 3 | 2 |
| 0 | 5 | 2 | 3 | 1 | 0 | 0 | 3 | 1 |
| 0 | 5 | 2 | 3 | 2 | 0 | 1 | 0 | 0 |
| 0 | 5 | 2 | 4 | 1 | 0 | 0 | 1 | 0 |
| 0 | 5 | 2 | 5 | 1 | 0 | 0 | 0 | 1 |
| 1 | 1 | 1 | 1 | 1 | 1 | 0 | 0 | 1 |
| 1 | 1 | 1 | 2 | 1 | 0 | 0 | 0 | 1 |
| 1 | 1 | 1 | 2 | 2 | 1 | 0 | 0 | 0 |
| 1 | 1 | 1 | 3 | 1 | 9 | 0 | 0 | 6 |
| 1 | 1 | 1 | 3 | 2 | 5 | 0 | 0 | 1 |
| 1 | 1 | 1 | 3 | 3 | 2 | 0 | 0 | 0 |
| 1 | 1 | 1 | 3 | 4 | 1 | 1 | 0 | 0 |
| 1 | 1 | 1 | 4 | 1 | 6 | 1 | 0 | 8 |
| 1 | 1 | 1 | 4 | 2 | 2 | 1 | 0 | 0 |
| 1 | 1 | 1 | 4 | 3 | 1 | 1 | 0 | 0 |
| 1 | 1 | 1 | 4 | 4 | 1 | 0 | 0 | 0 |
| 1 | 1 | 1 | 5 | 1 | 1 | 0 | 0 | 3 |
| 1 | 1 | 1 | 5 | 2 | 2 | 0 | 0 | 2 |
| 1 | 1 | 1 | 5 | 3 | 3 | 0 | 0 | 0 |
| 1 | 1 | 1 | 5 | 4 | 1 | 0 | 0 | 0 |
| 1 | 1 | 1 | 6 | 1 | 1 | 1 | 0 | 0 |
| 1 | 1 | 1 | 6 | 2 | 0 | 2 | 0 | 0 |
| 1 | 1 | 1 | 6 | 4 | 2 | 3 | 0 | 0 |
| 1 | 1 | 2 | 1 | 1 | 0 | 0 | 3 | 3 |
| 1 | 1 | 2 | 2 | 1 | 2 | 0 | 8 | 1 |
| 1 | 1 | 2 | 3 | 1 | 0 | 3 | 12 | 1 |
| 1 | 1 | 2 | 3 | 2 | 0 | 0 | 1 | 0 |
| 1 | 1 | 2 | 4 | 1 | 3 | 2 | 16 | 1 |
| 1 | 1 | 2 | 4 | 2 | 2 | 0 | 0 | 0 |
| 1 | 1 | 2 | 5 | 1 | 0 | 0 | 12 | 1 |
| 1 | 1 | 2 | 5 | 2 | 2 | 0 | 0 | 0 |
| 1 | 1 | 2 | 6 | 1 | 0 | 0 | 1 | 0 |
| 1 | 1 | 2 | 6 | 3 | 1 | 0 | 0 | 0 |
| 1 | 2 | 1 | 1 | 1 | 0 | 0 | 0 | 4 |
| 1 | 2 | 1 | 2 | 1 | 0 | 1 | 0 | 3 |
| 1 | 2 | 1 | 2 | 3 | 0 | 1 | 0 | 0 |
| 1 | 2 | 1 | 3 | 1 | 3 | 1 | 0 | 3 |
| 1 | 2 | 1 | 3 | 2 | 0 | 1 | 0 | 0 |
| 1 | 2 | 1 | 3 | 3 | 1 | 0 | 0 | 0 |
| 1 | 2 | 1 | 4 | 1 | 4 | 2 | 1 | 3 |
| 1 | 2 | 1 | 4 | 2 | 3 | 0 | 1 | 1 |
| 1 | 2 | 1 | 4 | 3 | 0 | 1 | 0 | 0 |
| 1 | 2 | 1 | 5 | 1 | 1 | 0 | 1 | 4 |
| 1 | 2 | 1 | 5 | 2 | 0 | 0 | 0 | 1 |
| 1 | 2 | 1 | 6 | 2 | 0 | 3 | 0 | 0 |
| 1 | 2 | 1 | 6 | 3 | 0 | 1 | 0 | 1 |
| 1 | 2 | 1 | 6 | 4 | 1 | 1 | 0 | 0 |
| 1 | 2 | 1 | 7 | 1 | 1 | 0 | 0 | 0 |
| 1 | 2 | 1 | 7 | 4 | 0 | 1 | 0 | 0 |
| 1 | 2 | 2 | 1 | 1 | 0 | 0 | 8 | 1 |
| 1 | 2 | 2 | 1 | 2 | 0 | 0 | 1 | 0 |
| 1 | 2 | 2 | 2 | 1 | 1 | 0 | 10 | 1 |
| 1 | 2 | 2 | 3 | 1 | 1 | 0 | 9 | 1 |
| 1 | 2 | 2 | 3 | 2 | 0 | 0 | 1 | 0 |
| 1 | 2 | 2 | 4 | 1 | 2 | 0 | 12 | 2 |
| 1 | 2 | 2 | 4 | 2 | 0 | 0 | 1 | 0 |
| 1 | 2 | 2 | 5 | 2 | 0 | 0 | 1 | 0 |
| 1 | 2 | 2 | 6 | 1 | 0 | 0 | 1 | 0 |
| 1 | 2 | 2 | 6 | 3 | 0 | 1 | 0 | 0 |
| 1 | 2 | 2 | 6 | 4 | 0 | 1 | 0 | 0 |
| 1 | 3 | 1 | 1 | 1 | 1 | 0 | 0 | 4 |
| 1 | 3 | 1 | 2 | 1 | 1 | 0 | 0 | 5 |
| 1 | 3 | 1 | 3 | 1 | 2 | 0 | 0 | 5 |
| 1 | 3 | 1 | 3 | 2 | 1 | 0 | 0 | 1 |
| 1 | 3 | 1 | 4 | 1 | 0 | 2 | 0 | 8 |
| 1 | 3 | 1 | 4 | 2 | 0 | 1 | 0 | 0 |
| 1 | 3 | 1 | 4 | 3 | 0 | 1 | 0 | 0 |
| 1 | 3 | 1 | 5 | 1 | 0 | 0 | 0 | 2 |
| 1 | 3 | 1 | 5 | 2 | 0 | 2 | 0 | 0 |
| 1 | 3 | 1 | 6 | 2 | 1 | 1 | 0 | 0 |
| 1 | 3 | 1 | 6 | 4 | 0 | 1 | 0 | 0 |
| 1 | 3 | 2 | 1 | 1 | 0 | 0 | 6 | 3 |
| 1 | 3 | 2 | 2 | 1 | 0 | 0 | 5 | 2 |
| 1 | 3 | 2 | 3 | 1 | 1 | 0 | 4 | 1 |
| 1 | 3 | 2 | 4 | 1 | 0 | 0 | 3 | 0 |
| 1 | 3 | 2 | 5 | 1 | 0 | 0 | 2 | 0 |
| 1 | 3 | 2 | 6 | 3 | 0 | 2 | 0 | 0 |
| 1 | 3 | 2 | 7 | 4 | 0 | 1 | 0 | 0 |
| 1 | 4 | 1 | 1 | 1 | 0 | 0 | 0 | 4 |
| 1 | 4 | 1 | 2 | 1 | 1 | 0 | 0 | 3 |
| 1 | 4 | 1 | 2 | 3 | 0 | 1 | 0 | 0 |
| 1 | 4 | 1 | 3 | 1 | 0 | 1 | 1 | 0 |
| 1 | 4 | 1 | 3 | 2 | 1 | 0 | 0 | 0 |
| 1 | 4 | 1 | 3 | 3 | 0 | 2 | 0 | 0 |
| 1 | 4 | 1 | 4 | 1 | 1 | 0 | 0 | 3 |
| 1 | 4 | 1 | 4 | 2 | 0 | 2 | 0 | 0 |
| 1 | 4 | 1 | 4 | 3 | 0 | 1 | 0 | 0 |
| 1 | 4 | 1 | 4 | 4 | 0 | 1 | 0 | 0 |
| 1 | 4 | 1 | 5 | 2 | 0 | 1 | 0 | 0 |
| 1 | 4 | 1 | 6 | 2 | 0 | 1 | 0 | 0 |
| 1 | 4 | 1 | 6 | 4 | 0 | 1 | 0 | 0 |
| 1 | 4 | 2 | 1 | 1 | 0 | 0 | 8 | 4 |
| 1 | 4 | 2 | 2 | 1 | 0 | 0 | 4 | 2 |
| 1 | 4 | 2 | 3 | 1 | 0 | 0 | 2 | 1 |
| 1 | 4 | 2 | 3 | 2 | 0 | 0 | 1 | 0 |
| 1 | 4 | 2 | 4 | 1 | 0 | 0 | 1 | 1 |
| 1 | 4 | 2 | 5 | 1 | 0 | 0 | 0 | 1 |
| 1 | 5 | 1 | 1 | 1 | 0 | 0 | 0 | 6 |
| 1 | 5 | 1 | 2 | 1 | 1 | 0 | 0 | 5 |
| 1 | 5 | 1 | 3 | 1 | 0 | 0 | 0 | 1 |
| 1 | 5 | 1 | 4 | 3 | 0 | 2 | 0 | 0 |
| 1 | 5 | 1 | 5 | 1 | 0 | 0 | 0 | 1 |
| 1 | 5 | 2 | 1 | 1 | 0 | 0 | 7 | 8 |
| 1 | 5 | 2 | 2 | 1 | 0 | 0 | 3 | 3 |
| 1 | 5 | 2 | 3 | 1 | 0 | 1 | 2 | 2 |
| 1 | 5 | 2 | 4 | 1 | 0 | 1 | 0 | 0 |
| 1 | 5 | 2 | 7 | 1 | 0 | 0 | 0 | 1 |

# Supplementary Table S2. Variable Inventory Used for Clustering Analysis

| Variable Name | Description | Measurement Scale | Source | Mean | SD |
| --- | --- | --- | --- | --- | --- |
| 1. Age | Survey indicator related to digital health literacy, barriers, or functional difficulty | Likert-scale / Ordinal | Digital Health Survey (Primary data) | 2.329 | 1.335 |
| 2. Gender | Survey indicator related to digital health literacy, barriers, or functional difficulty | Likert-scale / Ordinal | Digital Health Survey (Primary data) | 1.526 | 0.5 |
| 3. Education | Survey indicator related to digital health literacy, barriers, or functional difficulty | Likert-scale / Ordinal | Digital Health Survey (Primary data) | 3.218 | 1.545 |
| 4. Employment | Survey indicator related to digital health literacy, barriers, or functional difficulty | Likert-scale / Ordinal | Digital Health Survey (Primary data) | 2.746 | 1.083 |
| 5. Living status | Survey indicator related to digital health literacy, barriers, or functional difficulty | Likert-scale / Ordinal | Digital Health Survey (Primary data) | 2.027 | 0.552 |
| 6. Monthly Income Level | Survey indicator related to digital health literacy, barriers, or functional difficulty | Likert-scale / Ordinal | Digital Health Survey (Primary data) | 1.368 | 0.781 |
| 7. Are you familiar with the usage of smart phone | Survey indicator related to digital health literacy, barriers, or functional difficulty | Likert-scale / Ordinal | Digital Health Survey (Primary data) | 1.434 | 0.496 |
| 8. Are you familiar with the usage of Computer | Survey indicator related to digital health literacy, barriers, or functional difficulty | Likert-scale / Ordinal | Digital Health Survey (Primary data) | 1.893 | 0.309 |
| 9. If yes, how confident are you in using digital devices | Survey indicator related to digital health literacy, barriers, or functional difficulty | Likert-scale / Ordinal | Digital Health Survey (Primary data) | 2.464 | 0.922 |
| 10. If no, what is your mode of gathering information from internet | Survey indicator related to digital health literacy, barriers, or functional difficulty | Likert-scale / Ordinal | Digital Health Survey (Primary data) | 3.268 | 1.272 |
| 11. How much is your level of understanding of English language | Survey indicator related to digital health literacy, barriers, or functional difficulty | Likert-scale / Ordinal | Digital Health Survey (Primary data) | 1.62 | 0.986 |
| 12. Are you aware that vernacular language options are available in internet | Survey indicator related to digital health literacy, barriers, or functional difficulty | Likert-scale / Ordinal | Digital Health Survey (Primary data) | 1.455 | 0.498 |
| 13. Do you have reliable internet access | Survey indicator related to digital health literacy, barriers, or functional difficulty | Likert-scale / Ordinal | Digital Health Survey (Primary data) | 1.47 | 0.499 |
| 14. Are you aware of the availability of health-related information in internet | Survey indicator related to digital health literacy, barriers, or functional difficulty | Likert-scale / Ordinal | Digital Health Survey (Primary data) | 1.569 | 0.496 |
| 15. If yes, do you search internet for gathering health-related information? | Survey indicator related to digital health literacy, barriers, or functional difficulty | Likert-scale / Ordinal | Digital Health Survey (Primary data) | 1.514 | 0.5 |
| 16. If yes how often do you use internet for gathering health related information. | Survey indicator related to digital health literacy, barriers, or functional difficulty | Likert-scale / Ordinal | Digital Health Survey (Primary data) | 2.901 | 1.275 |
| 17. Do you face any difficulties in understanding or using digital health tools | Survey indicator related to digital health literacy, barriers, or functional difficulty | Likert-scale / Ordinal | Digital Health Survey (Primary data) | 1.94 | 0.71 |
| Teleconsultation | Survey indicator related to digital health literacy, barriers, or functional difficulty | Likert-scale / Ordinal | Digital Health Survey (Primary data) | 1.421 | 0.606 |
| Telemedicine | Survey indicator related to digital health literacy, barriers, or functional difficulty | Likert-scale / Ordinal | Digital Health Survey (Primary data) | 1.582 | 0.833 |
| Wearable devices | Survey indicator related to digital health literacy, barriers, or functional difficulty | Likert-scale / Ordinal | Digital Health Survey (Primary data) | 1.282 | 0.697 |
| Self-monitoring devices | Survey indicator related to digital health literacy, barriers, or functional difficulty | Likert-scale / Ordinal | Digital Health Survey (Primary data) | 1.879 | 1.222 |
| Mobile health apps | Survey indicator related to digital health literacy, barriers, or functional difficulty | Likert-scale / Ordinal | Digital Health Survey (Primary data) | 1.299 | 0.776 |
| Government initiatives | Survey indicator related to digital health literacy, barriers, or functional difficulty | Likert-scale / Ordinal | Digital Health Survey (Primary data) | 1.646 | 0.82 |
| 18.1. In a usual week, on how many days do you engage in at least 30 minutes of physcial activity? | Survey indicator related to digital health literacy, barriers, or functional difficulty | Likert-scale / Ordinal | Digital Health Survey (Primary data) | 2.371 | 1.152 |
| 18. 2. On those days, what is your average total time per day spent in physical activity? | Survey indicator related to digital health literacy, barriers, or functional difficulty | Likert-scale / Ordinal | Digital Health Survey (Primary data) | 2.286 | 1.172 |
| 18.3. How would you rate your overall physical activity level over the past month? | Survey indicator related to digital health literacy, barriers, or functional difficulty | Likert-scale / Ordinal | Digital Health Survey (Primary data) | 2.472 | 1.151 |
| 19. What are the main barriers you face when using technology for health purposes? | Survey indicator related to digital health literacy, barriers, or functional difficulty | Likert-scale / Ordinal | Digital Health Survey (Primary data) | 2.278 | 1.396 |
| 20. How would you describe your overall health? | Survey indicator related to digital health literacy, barriers, or functional difficulty | Likert-scale / Ordinal | Digital Health Survey (Primary data) | 3.731 | 0.974 |
| 22. How often do you require medical consultations? | Survey indicator related to digital health literacy, barriers, or functional difficulty | Likert-scale / Ordinal | Digital Health Survey (Primary data) | 2.534 | 1.054 |
| 23. Do you feel digital health services have improved your health outcomes? | Survey indicator related to digital health literacy, barriers, or functional difficulty | Likert-scale / Ordinal | Digital Health Survey (Primary data) | 1.979 | 0.71 |
| 24. The cost of digital consultation is higher than the traditional methods | Survey indicator related to digital health literacy, barriers, or functional difficulty | Likert-scale / Ordinal | Digital Health Survey (Primary data) | 2.312 | 0.899 |
| 25. Do you think that cost of digital material on health is higher than the print health material | Survey indicator related to digital health literacy, barriers, or functional difficulty | Likert-scale / Ordinal | Digital Health Survey (Primary data) | 2.32 | 0.896 |
| 1. I can easily click on links and buttons while using health websites. | Survey indicator related to digital health literacy, barriers, or functional difficulty | Likert-scale / Ordinal | Digital Health Survey (Primary data) | 2.688 | 1.531 |
| 2. I am confident in choosing the most relevant health information from online search results. | Survey indicator related to digital health literacy, barriers, or functional difficulty | Likert-scale / Ordinal | Digital Health Survey (Primary data) | 2.689 | 1.521 |
| 3. I know how to use the right search words to find the health information I need. | Survey indicator related to digital health literacy, barriers, or functional difficulty | Likert-scale / Ordinal | Digital Health Survey (Primary data) | 2.69 | 1.518 |
| 4. I am able to find specific health information that I am looking for on the internet. | Survey indicator related to digital health literacy, barriers, or functional difficulty | Likert-scale / Ordinal | Digital Health Survey (Primary data) | 2.684 | 1.516 |
| 5. I can tell if the health information I find online is reliable or not. | Survey indicator related to digital health literacy, barriers, or functional difficulty | Likert-scale / Ordinal | Digital Health Survey (Primary data) | 2.682 | 1.516 |
| 6. I can identify if the health information online is influenced by commercial interests | Survey indicator related to digital health literacy, barriers, or functional difficulty | Likert-scale / Ordinal | Digital Health Survey (Primary data) | 2.649 | 1.5 |
| 7. I compare health information across different websites to check for consistency. | Survey indicator related to digital health literacy, barriers, or functional difficulty | Likert-scale / Ordinal | Digital Health Survey (Primary data) | 2.52 | 1.418 |
| 8. I can decide if the health information I find online applies to my personal health situation. | Survey indicator related to digital health literacy, barriers, or functional difficulty | Likert-scale / Ordinal | Digital Health Survey (Primary data) | 2.439 | 1.327 |
| 9. I feel confident in using the health information I find online to improve my daily health practices. | Survey indicator related to digital health literacy, barriers, or functional difficulty | Likert-scale / Ordinal | Digital Health Survey (Primary data) | 2.449 | 1.305 |
| 10. use online health information to make informed decisions about my health- on medication, | Survey indicator related to digital health literacy, barriers, or functional difficulty | Likert-scale / Ordinal | Digital Health Survey (Primary data) | 2.542 | 1.398 |
| 11. I rarely get confused while navigating health websites | Survey indicator related to digital health literacy, barriers, or functional difficulty | Likert-scale / Ordinal | Digital Health Survey (Primary data) | 2.708 | 1.564 |
| 12. I know how to return to a previous webpage if I lost my way on a health website. | Survey indicator related to digital health literacy, barriers, or functional difficulty | Likert-scale / Ordinal | Digital Health Survey (Primary data) | 2.588 | 1.462 |
| 13. I rarely click on things by mistake while searching for health information online. | Survey indicator related to digital health literacy, barriers, or functional difficulty | Likert-scale / Ordinal | Digital Health Survey (Primary data) | 2.33 | 1.21 |
| 14. I can clearly express my health concerns when communicating online to a doctor | Survey indicator related to digital health literacy, barriers, or functional difficulty | Likert-scale / Ordinal | Digital Health Survey (Primary data) | 2.212 | 1.087 |
| 15. I am confident about deciding whether the health information I find online is accurate. | Survey indicator related to digital health literacy, barriers, or functional difficulty | Likert-scale / Ordinal | Digital Health Survey (Primary data) | 2.051 | 1.019 |
| 16. I am competent at deciding between trustworthy and untrustworthy health websites. | Survey indicator related to digital health literacy, barriers, or functional difficulty | Likert-scale / Ordinal | Digital Health Survey (Primary data) | 2.126 | 1.143 |
| 1. I have difficulties in connecting to the internet, preventing me from using online health services. | Survey indicator related to digital health literacy, barriers, or functional difficulty | Likert-scale / Ordinal | Digital Health Survey (Primary data) | 3.442 | 0.622 |
| 2. The design of digital health platforms is not user-friendly for the elderly. | Survey indicator related to digital health literacy, barriers, or functional difficulty | Likert-scale / Ordinal | Digital Health Survey (Primary data) | 4.715 | 0.574 |
| 3. The guidelines provided for digital health services are too hard for me to understand. | Survey indicator related to digital health literacy, barriers, or functional difficulty | Likert-scale / Ordinal | Digital Health Survey (Primary data) | 3.862 | 0.496 |
| 4. The font size and display settings on health applications and webpages are too small or unclear for me. | Survey indicator related to digital health literacy, barriers, or functional difficulty | Likert-scale / Ordinal | Digital Health Survey (Primary data) | 3.249 | 1.406 |
| 5. I am concerned about making mistakes while using digital health services, such as entering inaccurate information. | Survey indicator related to digital health literacy, barriers, or functional difficulty | Likert-scale / Ordinal | Digital Health Survey (Primary data) | 3.812 | 0.65 |
| 6. I frequently experience anxiety or worry when attempting to use internet health services | Survey indicator related to digital health literacy, barriers, or functional difficulty | Likert-scale / Ordinal | Digital Health Survey (Primary data) | 3.756 | 0.786 |
| 7. I don't believe the information offered by digital health platforms | Survey indicator related to digital health literacy, barriers, or functional difficulty | Likert-scale / Ordinal | Digital Health Survey (Primary data) | 4.037 | 0.968 |
| 8. I worry that my personal health information could be misused or accessed by unauthorized people | Survey indicator related to digital health literacy, barriers, or functional difficulty | Likert-scale / Ordinal | Digital Health Survey (Primary data) | 4.66 | 0.678 |
| 9. I cannot find a need to use digital health services because I prefer spending time with healthcare practitioners | Survey indicator related to digital health literacy, barriers, or functional difficulty | Likert-scale / Ordinal | Digital Health Survey (Primary data) | 4.542 | 0.815 |
| 10. The health services provided online do not address my health problems or needs. | Survey indicator related to digital health literacy, barriers, or functional difficulty | Likert-scale / Ordinal | Digital Health Survey (Primary data) | 3.418 | 0.741 |
| 11. I prefer to seek health advice from family and friends rather than using technology services. | Survey indicator related to digital health literacy, barriers, or functional difficulty | Likert-scale / Ordinal | Digital Health Survey (Primary data) | 4.082 | 0.605 |
| 12. I lack the necessary assistance and training to effectively use digital health platforms. | Survey indicator related to digital health literacy, barriers, or functional difficulty | Likert-scale / Ordinal | Digital Health Survey (Primary data) | 4.721 | 0.569 |
| 13. It is challenging to find someone who can help me to access digital health services when I need it. | Survey indicator related to digital health literacy, barriers, or functional difficulty | Likert-scale / Ordinal | Digital Health Survey (Primary data) | 4.658 | 0.603 |
| 14. Do you feel that there is enough government support for the adoption of digital health services among the elderly population | Survey indicator related to digital health literacy, barriers, or functional difficulty | Likert-scale / Ordinal | Digital Health Survey (Primary data) | 4.485 | 0.639 |
| 15. I seldom experience difficulty understanding the medical terminology used in internet health information | Survey indicator related to digital health literacy, barriers, or functional difficulty | Likert-scale / Ordinal | Digital Health Survey (Primary data) | 4.448 | 0.608 |

**Supplementary Table S3. Comparative Summary of Key Variables Across Clusters**

| Variable | Low-Risk (n=440) | High-Risk (n=360) | p-value |
| --- | --- | --- | --- |
|  | Mean ± SD | Mean ± SD | (Mann-Whitney U) |

Digital Health Literacy

| Variable | Low-Risk (n=440) | High-Risk (n=360) | p-value |
| --- | --- | --- | --- |
| Digital Health Literacy Score (Composite) | 3.77 ± 0.51 | 1.24 ± 0.35 | <0.001 |
| 1. I can easily click on links and buttons while using health websites | 3.94 ± 0.74 | 1.10 ± 0.30 | <0.001 |
| 2. I am confident in choosing the most relevant health information | 3.86 ± 0.67 | 1.13 ± 0.34 | <0.001 |
| 3. I know how to use the right search words to find health information | 3.87 ± 0.68 | 1.13 ± 0.34 | <0.001 |
| 4. I am able to find specific health information on the internet | 3.87 ± 0.67 | 1.13 ± 0.34 | <0.001 |
| 5. I can tell if the health information I find online is reliable | 3.77 ± 0.77 | 1.15 ± 0.36 | <0.001 |
| 6. I can identify if health information is influenced by commercial interests | 3.81 ± 0.74 | 1.14 ± 0.35 | <0.001 |
| 7. I compare health information across different websites | 3.78 ± 0.76 | 1.14 ± 0.35 | <0.001 |
| 8. I can decide if health information applies to my personal situation | 3.60 ± 0.82 | 1.17 ± 0.38 | <0.001 |
| 9. I feel confident using health information to improve daily practices | 3.34 ± 0.83 | 1.16 ± 0.37 | <0.001 |
| 10. I use online health information for informed decisions about medication | 3.28 ± 0.79 | 1.16 ± 0.37 | <0.001 |
| 11. I rarely get confused while navigating health websites | 3.61 ± 0.87 | 1.18 ± 0.39 | <0.001 |
| 12. I know how to return to a previous webpage if lost | 4.07 ± 0.67 | 1.26 ± 0.44 | <0.001 |
| 13. I rarely click on things by mistake while searching | 3.58 ± 0.82 | 1.35 ± 0.48 | <0.001 |
| 14. I can clearly express health concerns when communicating online | 3.46 ± 0.80 | 1.38 ± 0.49 | <0.001 |
| 15. I am confident about deciding if health information is accurate | 3.71 ± 0.74 | 1.37 ± 0.48 | <0.001 |
| 16. I am competent at deciding between trustworthy and untrustworthy websites | 3.67 ± 0.75 | 1.37 ± 0.48 | <0.001 |

Functional Difficulty

| Variable | Low-Risk (n=440) | High-Risk (n=360) | p-value |
| --- | --- | --- | --- |
| Functional Difficulty Score (Composite) | 4.01 ± 0.32 | 4.15 ± 0.37 | <0.001 |
| 1. Difficulties connecting to the internet | 3.61 ± 0.59 | 3.24 ± 0.60 | <0.001 |
| 2. Digital health platforms not user-friendly for elderly | 4.66 ± 0.54 | 4.78 ± 0.61 | <0.001 |
| 3. Guidelines for digital health services too hard to understand | 3.77 ± 0.51 | 3.97 ± 0.46 | <0.001 |
| 4. Font size and display settings too small or unclear | 3.18 ± 1.42 | 3.33 ± 1.39 | 0.124 |
| 5. Concerned about making mistakes using digital health services | 3.67 ± 0.70 | 3.98 ± 0.53 | <0.001 |
| 6. Frequently experience anxiety when using internet health services | 3.55 ± 0.86 | 4.01 ± 0.59 | <0.001 |
| 7. Don't believe information offered by digital health platforms | 3.62 ± 0.88 | 4.55 ± 0.81 | <0.001 |
| 8. Worry personal health information could be misused | 4.59 ± 0.68 | 4.74 ± 0.67 | <0.001 |
| 9. Cannot find a need for digital health services (prefer practitioners) | 4.60 ± 0.77 | 4.47 ± 0.87 | 0.037 |
| 10. Online health services do not address my health problems | 3.38 ± 0.70 | 3.46 ± 0.79 | 0.150 |
| 11. Prefer to seek health advice from family and friends | 4.09 ± 0.55 | 4.08 ± 0.66 | 0.933 |
| 12. Lack necessary assistance and training for digital health platforms | 4.76 ± 0.49 | 4.67 ± 0.65 | 0.123 |
| 13. Challenging to find someone to help access digital health services | 4.66 ± 0.55 | 4.65 ± 0.66 | 0.465 |

Self-Management Capacity

| Variable | Low-Risk (n=440) | High-Risk (n=360) | p-value |
| --- | --- | --- | --- |
| Difficulties in understanding or using digital health tools | 2.08 ± 0.79 | 1.76 ± 0.56 | <0.001 |
| Digital health services have improved health outcomes | 1.85 ± 0.74 | 2.13 ± 0.64 | <0.001 |

Trust in Online Health Information

| Variable | Low-Risk (n=440) | High-Risk (n=360) | p-value |
| --- | --- | --- | --- |
| Don't believe information offered by digital health platforms | 3.62 ± 0.88 | 4.55 ± 0.81 | <0.001 |
| Enough government support for digital health adoption among elderly | 4.67 ± 0.57 | 4.26 ± 0.64 | <0.001 |
| Seldom experience difficulty understanding medical terminology | 4.72 ± 0.52 | 4.11 ± 0.53 | <0.001 |

Health Indicators

| Variable | Low-Risk (n=440) | High-Risk (n=360) | p-value |
| --- | --- | --- | --- |
| Overall physical activity level (past month) | 2.42 ± 1.11 | 2.53 ± 1.19 | 0.304 |
| Overall health self-rating | 3.76 ± 0.93 | 3.70 ± 1.02 | 0.715 |
| Chronic illness or long-term health issues | 4.77 ± 2.68 | 5.05 ± 2.52 | 0.293 |
| Frequency of medical consultations | 2.52 ± 1.02 | 2.56 ± 1.09 | 0.718 |
